# Bowman-Birk Inhibitors: Insights into Family of Multifunctional Proteins and Peptides with Potential Therapeutical Applications

**DOI:** 10.3390/ph13120421

**Published:** 2020-11-25

**Authors:** Agata Gitlin-Domagalska, Aleksandra Maciejewska, Dawid Dębowski

**Affiliations:** Department of Molecular Biochemistry, Faculty of Chemistry, University of Gdansk, Wita Stwosza 63, 80-308 Gdansk, Poland; agata.domagalska@ug.edu.pl (A.G.-D.); aleksandra.d.maciejewska@gmail.com (A.M.)

**Keywords:** Bowman-Birk inhibitors, protease inhibitors, plant-derived inhibitors, amphibian-derived inhibitors, legumes, chemopreventive, anti-inflammatory, antimicrobial

## Abstract

Bowman-Birk inhibitors (BBIs) are found primarily in seeds of legumes and in cereal grains. These canonical inhibitors share a highly conserved nine-amino acids binding loop motif CTP1SXPPXC (where P1 is the inhibitory active site, while X stands for various amino acids). They are natural controllers of plants’ endogenous proteases, but they are also inhibitors of exogenous proteases present in microbials and insects. They are considered as plants’ protective agents, as their elevated levels are observed during injury, presence of pathogens, or abiotic stress, i.a. Similar properties are observed for peptides isolated from amphibians’ skin containing 11-amino acids disulfide-bridged loop CWTP1SXPPXPC. They are classified as Bowman-Birk like trypsin inhibitors (BBLTIs). These inhibitors are resistant to proteolysis and not toxic, and they are reported to be beneficial in the treatment of various pathological states. In this review, we summarize up-to-date research results regarding BBIs’ and BBLTIs’ inhibitory activity, immunomodulatory and anti-inflammatory activity, antimicrobial and insecticidal strength, as well as chemopreventive properties.

## 1. Introduction

The complete set of proteases in an organism, known as a human degradome, is encoded by over 550 genes and represents more than 2% of the whole human genome [1]. Although proteases are exclusively specialized in the hydrolysis of peptide bonds, they are classified into several groups, applying different modes of action: metalloproteases (the most abundant), serine proteases, cysteine proteases, aspartyl proteases, threonine proteases, glutamic proteases, and asparagine lyases [2]. Proteases are implicated in numerous key biological processes, such as cell development and apoptosis, tissue modeling, angiogenesis, blood coagulation, wound healing, protein turnover, zymogen activation, and regulation of signaling cascades. Their dysregulated activity can bring destructive effects, as reported for various disorders, including cancers [3], inflammatory [4], and cardiovascular diseases [5]. Notably, there are more than 130 hereditary diseases related to mutations in proteases’ genes [6].

The proteolytic activity has to be tightly regulated at multiple stages, starting with transcription. In order to control proteases action, sophisticated mechanisms are utilized, including posttranslational modifications, production of inactive zymogens, and their rational conversion into active forms, as well as binding of enzymes with endogenous inhibitors [7,8].

The protease inhibitors offer high pharmaceutical potential in the treatment of diseases in which upregulated proteolytic activity is observed. Drugbank online database, which gathers drug compounds approved by the U.S. Food and Drug Administration (FDA), provides data concerning 108 natural and synthetic protease inhibitors [9]. Peptide and protein-based inhibitors are divided into families based on their primary and three-dimensional structures and mechanisms of inhibition [10]. According to the comprehensive MEROPS database [2], protease inhibitors are classified into 38 clans and subdivided into 78 families. Among them, various families of inhibitors, including serpins, phytocystatins, Kunitz-type inhibitors (KTIs), Bowman-Birk inhibitors (BBIs), bifunctional α-amylase-trypsin inhibitors, mustard-type inhibitors, potato type-I and potato type-II inhibitors, potato metallocarboxypeptidase inhibitors, and squash and cyclotide inhibitors have gained much attention recently [11]. This is mostly due to their potential application in the treatment of neurodegenerative disease, cancer, and autoimmune disorders [11,12,13,14,15]. Such inhibitors are found mostly in seeds, leaves, and tubers of plants. They are supposed to regulate the activity of both endogenous proteases and exogenous digestive enzymes produced by phytopathogens. Thus, plant-derived inhibitors are considered as plant defense system components. Moreover, they are also regarded as storage of sulfur-containing amino acids.

Two major clusters of these inhibitors are KTIs and BBIs families. The main difference between their members is the number of disulfide linkages—BBIs contain usually seven, while most KTIs two disulfide bonds. KTIs contain a single reactive site, while, in some BBIs, there are two reactive sites. Interestingly, both families share a similar mechanism of inhibition [16]. They are found in legumes; some plants contain members of both families, while, in others, only one of them occurs, e.g., BBIs are present exclusively in common bean and lentil. BBIs are found primarily in the seeds of legumes and in cereal grains.

## 2. Bowman-Birk Inhibitors (BBIs)

According to the MEROPS database, BBIs are coded as I12 (holotype: Bowman-Birk trypsin/chymotrypsin inhibitor unit 1) and I99 (holotype: Bowman-Birk-like trypsin inhibitor; *Odorrana versabilis*) [2]. According to Hellinger and Gruber [11], there are 611 BBIs out of 6720 identified inhibitors in plants, which account for 9.1%. The phrase “Bowman-Birk serine protease inhibitor family” (used without additional restrictions) results in 49 reviewed and 779 unreviewed records in the web database www.uniprot.org, which collects comprehensive protein sequence and functional information.

The first representative of the BBI family was isolated from soybean (*Glycine max*) by Donald E. Bowman in 1946 [17] and further characterized in 1963 by Yehudith Birk et al. [18]. Currently, it is often referred to as ‘classical BBI’ (here, it is abbreviated as BBI). BBIs are usually isolated from plants using multi-step chromatographic procedures [19]. Interestingly, Fields et al. [20] proposed a novel concept based on high gradient magnetic separation and synthetic dodecapeptides, identified by phage display technology, targeting specifically BBI. Upon immobilization on superparamagnetic microbeads, the selected peptides were able to bind and isolate BBI from crude soy whey extracts. Palavalli et al. [21] demonstrated that active BBI and other proteins might be released into the surrounding media from seeds upon 4-8 h incubation in the water at 50 °C.

BBIs are one of the best recognized and characterized natural protease inhibitors family, as evidenced by the presence of several comprehensive review articles [13,14,22,23]. However, their role in plants is not unequivocally defined. Their elevated expression is observed in various situations considered dangerous for plants, such as injury, the presence of fungus and pathogens, or abiotic stress. BBIs are likely involved in the development of salt [24] and drought [25] stress tolerance. The latter function may be associated with a decrease of drought-induced oxidative stress [26]. The higher concentration of two homologs BBIs (with 87% similarity in their amino acid sequences) has also been observed in rice under Fe-deficient conditions [27]. They both interact with transcription factor IDEF1 (iron deficiency-responsive cis-acting element binding factor), which plays a crucial role in iron deficiency-induced gene regulation involved in iron homeostasis, and prevents its 26S proteasome-dependent degradation [27].

BBIs’ defensive function is reflected in an insecticidal activity, as various members of this family display antifeedant activity against insects. Thus, the transfer of the BBI gene into plants with economic importance is a promising strategy to produce transgenic plants resistant to insects [28]. Some BBIs are also blocking proteases produced by pathogens; thus, they have the potential to be used as antimicrobials [29]. As mentioned before, the BBIs family was established as a bunch of plant-derived inhibitors; however, a novel group of peptides originating from animals, which imitates the BBI’s trypsin inhibitory loop (TIL), has been recently identified [30,31,32]. These peptides were isolated from frogs’ skin, and similarly to plant BBIs, they present strong trypsin inhibitory activity. Their disulfide-bridged loop contains 11 residues, with the general formula CWTP1SX_1_PPX_2_PC (where P1 is the inhibitory active site usually occupied by Lys, while X_1_ and X_2_ are variable) [30,32]. This loop is longer than that found in plant BBIs composed of 9-amino acids (CTP1SX_1_PPX_2_C). Since the spatial structures of both binding loops are highly similar, although not identical, these trypsin inhibitors are termed as BBI-like trypsin inhibitors (here, abbreviated as BBLTIs).

In this review, the current knowledge about both plant-derived BBIs and amphibian-originating BBLTIs is summarized regarding their inhibitory potency, immunomodulatory and anti-inflammatory activity, antimicrobial and insecticidal strength, as well as chemopreventive properties.

## 3. Canonical Inhibitors (Standard Mechanism Inhibitors)

Canonical inhibitors (standard mechanism inhibitors) include peptides and proteins grouped in at least 19 convergently evolved families [33]. They are widely distributed in all forms of life and interact with target enzymes reversibly in a substrate-like manner [10,34]. Such a mode of action is known as ‘standard’ or Laskowski’s mechanism of inhibition. Despite differences in their sequences, canonical inhibitors share a strikingly similar and distinctive binding motif, called the protease-binding loop, which is described in detail elsewhere [35,36,37,38]. This conformationally conserved, fairly rigid, solvent-exposed, and extended loop is complementary to the concave active site found on the surface of the target enzyme(s). Its central part contains a peptide bond, described as a reactive P1-P1′ site [36]. According to the Schechter and Berger nomenclature [39], P1-P1′ is the scissile peptide bond, and both P1 and P1′ amino acid residues interact with the corresponding enzyme’s S1 and S1′ subsites, respectively. The interaction with enzymes is mainly regulated by the P1 residues; however, both—inhibitory activity and specificity—are also modulated by adjacent amino acids. Variations among amino acids found in the inhibitory region have been observed [40]. Some inhibitors contain a single binding loop, while others bear multiple domains with binding loops. The latter are able to interact with protease in other stoichiometry than 1:1 or inhibit more than one enzyme simultaneously [36].

In contrast to the substrates, canonical inhibitors bind to an enzyme more tightly, and hydrolysis of their scissile peptide bond is markedly slower, usually by factors of 10^6^–10^10^ [41]. The cleavage of the reactive site bond, which leads to the formation of acyl-enzyme complex, is rather fast, but the other steps (i.e., the release of a first cleaved product with the novel amino terminus, its replacement by a water molecule, and a formation of the second cleaved product with a new carboxyl terminus) proceed very slowly and limit the hydrolysis pace. The main forces involved in maintaining the conformation of the canonical loop, as well as prevention from hydrolysis of the reactive bond, are an extensive hydrogen-bonding network and disulfide bond(s) [35,36]. Their presence also promotes efficient resynthesis of the P1-P1′ bond. The newly formed, in the process of hydrolysis, *N*-terminus is oriented in an optimal position for nucleophilic attack on the acyl-enzyme, which leads to religation of the cleaved P1-P1′ bond. For instance, regarding sunflower (*Helianthus annuus*) trypsin inhibitor (SFTI-1), the ratio of native, uncleaved SFTI-1 to the cleaved SFTI-1 is approximately 9:1, regardless of which inhibitor’s form is incubated with trypsin [42]. The interaction between canonical inhibitor and protease, basing mostly on X-ray analyses, was initially explained by the lock-and-key model. However, a solution-state structural study revealed considerable flexibility in the protease binding loop and its conformational change upon binding to the enzyme [43].

## 4. BBIs Hallmarks

### 4.1. Mono- and Double-Headed Structure

Plant-derived BBIs from dicotyledonous usually have a low molecular weight between 6 and 9 kDa and two homologous and independent binding loops located at the opposite sites of the molecules. Such ‘double-headed’ inhibitors are capable of inhibiting two, the same or different, enzyme molecules either simultaneously or independently. In contrast, BBIs from monocotyledonous are more diverse and are divided into two subclasses containing either ‘mono-headed’ inhibitors with a molecular weight of about 8 kDa or ‘double-headed’ with molecular weight ~16 kDa. The latter inhibitors might have evolved from 8 kDa representatives by gene duplication [44]. In ‘double-headed’ inhibitors, the first binding loop is usually involved in the inhibition of trypsin, and its P1 position is occupied by Lys or Arg. The second inhibitory domain is mostly associated with chymotrypsin and contains hydrophobic amino acids, such as Phe, Tyr, Leu, or Trp [45]. Due to the presence of these inhibitory loops, BBIs exhibit a high affinity toward trypsin and chymotrypsin, as it is shown in Table 1. Interestingly, soybean classical BBI displays several exposed hydrophobic patches on its surface, whereas charged amino acids and water molecules are found in its interior. This feature makes BBI unusual, as standard globular proteins have a hydrophobic core and external polar residues [46]. Similar hydrophobic surface-exposed patches are also observed in some cyclotide family of circular inhibitors [47] and in cystine-knot miniproteins, also known as knottins [48]. The family of BBI contains also a strong trypsin inhibitor SFTI-1 composed of just 14 amino acids (~1.5 kDa) [49]. Even though this backbone-cyclized peptide containing a single disulfide bond is not genetically related to other BBIs [50], they share an almost identical binding loop [51]. It is worth noting that SFTI-1 is one of the most popular starting structures to produce potent inhibitors of a wide range of biologically relevant proteases (Table 2).

### 4.2. Self-Association of BBIs

BBIs are well-known to undergo spontaneous self-associations in solution to form homodimers, trimers, or even more complex multimeric forms [52,53,54,55,56,57,58,59,60,61]. Various forces contribute to the formation and stabilization of such oligomers. For example, an internal hydrogen-bonded network supported by sets of hydrophobic contacts has been reported in a dimer of trypsin/chymotrypsin inhibitor from winter pea seeds (*Pisum sativum*, PsTI-IVb) [59], while ion pair interaction between an *ε*-amino group of the P1 Lys24 residue found in one monomer and the carboxyl of the *C*-terminal Asp86 of the second monomer has been observed in the major inhibitor isolated from *Dolichos biflorus* (horsegram) HGI-III (horsegram inhibitor III) [54]. The tendency of black-eyed pea trypsin and chymotrypsin inhibitor (BTCI) to self-association and formation of oligomers has been demonstrated using light scattering [60], atomic force microscopy (AFM) combined with molecular modeling and docking studies [61], and surface plasmon resonance [58]. Based on the first method, BTCI forms a trimer at 15 µM and pH 7.5 with a hydrodynamic diameter of 4.5 nm [60]. The second attempt revealed that BTCI exists in monomer-dimer-trimer-hexamer equilibrium [61]. It is speculated that a more hydrophobic and flexible chymotrypsin-binding loop, with respect to the antitryptic subdomain, could be correlated to the self-aggregation tendency and oligomer formation [62,63]. The tendency to self-association is mostly related to the physiological function of BBIs as a plant storage protein since it facilitates their tight packing in seeds. It has been also hypothesized that amino acid residues involved in the self-association process might participate in intermolecular interactions of BBIs with other enzymes and macromolecules, which might be associated with anti-carcinogenic activity [64].

### 4.3. Presence of Isoforms

Different isoforms of BBIs are frequently present in the same plant [55,75,76,78,84,86,120,121,122]. It has been proposed that isoinhibitors are produced due to the co-evolution of the plants and insects [123]. Such a strategy is likely applied to increase efficiency in combating pathogens. This may minimize the risk of hydrolysis of all inhibitors by the pest enzymes as well as help to deal with inhibitor-insensitive or inhibitor-degrading proteinases [124,125]. It has been shown that isoinhibitors may be formed either via posttranslational proteolytic processing at *N*- and *C*-terminal regions of a primary gene product [126] or may be expressed by different genes [78,127,128]. For example, soy seeds contain a mixture of several BBI isoforms and their genetic variants, with two major components—IBB1 (Bowman-Birk proteinase inhibitor, Swiss-Prot accession number P01055) and IBBD2 (Bowman-Birk type proteinase inhibitor D-II, P01064)—having different protease inhibitory activities [66]. The presence of Lys and Leu in the P1 positions in binding loops of IBB1 confers specificity for the inhibition of trypsin (*K*_i_ of 29.8 nM) and chymotrypsin (*K*_i_ of 3.3 nM) activity. On the other hand, while the IBBD2 Arg residues found in the P1 position of both IBBD2 binding loops determine its antitrypsin activity (*K*_i_ of 14.8 nM).

### 4.4. Extreme Stability

The presence of several disulfide bonds makes BBIs extremely stable inhibitors, able to block enzymatic activity even after incubation at high temperatures (even about 100 °C) and a wide pH range (2–12) [55,78,129,130]. Indeed, IBB1 isoform has been reported to retain 75% of its activity after 360 min incubation at 100 °C. On the contrary, in similar conditions, the soy extract loses its ability to inhibit trypsin and chymotrypsin faster [120,131]. This shows that the thermal stability of a highly pure inhibitor differs from plant extract, which may result from the presence of other compounds in the extract, able to affect the inhibitor’s structural stability due to protein aggregation [132]. It has been shown that antichymotrypsin activity is inactivated faster than antitrypsin one, which is associated with the higher thermal stability of the trypsin-reactive subdomain. Moreover, heating causes gradual conformational changes of BBI, observed by far-UV and near-UV CD spectra. He et al. proposed a heat-induced inactivation mechanism of soybean BBI in which *β*-elimination reactions convert disulfide bonds into free SH and dehydroalanine, while serine into dehydroalanine. Subsequent intramolecular reactions between these novel species, as well as between generated dehydroalanine and lysine to produce lysinoalanine, may create novel cross-links in BBI [133].

The crucial role of disulfide bridges in the stabilization of BBIs’ reactive site conformation has been confirmed using dithiothreitol (DTT) or 2-mercaptoethanol. Both reagents reduce BBIs and usually lead to complete abolition of their trypsin and chymotrypsin inhibitory activities [14,121]. However, there are some exceptions, e.g., horsegram proteinase inhibitor (HGPI) retains 50% of its inhibitory activity against trypsin and chymotrypsin even after 90 min treatment with 1.0 mM DTT [134].

The classical BBI turns out to be highly resistant to the harsh acidic environment of the gastrointestinal (GI) tract and to the presence of proteolytic enzymes [121,135]. Notably, as was presented by Persiani et al. [136], a ^125^I labeled BBI was cleared from the blood 30 min after intravenous injection and was completely degraded within 24 h.

## 5. Biological Properties

### 5.1. Anticarcinogenic Activity

Chemopreventive activities of various BBIs, isolated from different plants, have been reviewed comprehensively several times [13,14,22,23,137]. Table 3 contains a brief summary of the most important reports concerning the anticarcinogenic activity of both—plant BBIs and animal BBLTIs.

Antiproliferative activities of BBIs are thought to be associated with their intrinsic ability to inhibit serine proteases. It has been hypothesized that strong inhibitory activity, induced particularly by chymotrypsin-binding site, is necessary to evoke effective anti-carcinogenic actions [170,171]. Therefore, the potencies of both—pure BBI and a soybean-derived extract enriched in BBI (known as BBIC)—are sometimes expressed in chymotrypsin inhibitor units (CIU). One CIU is the amount of a substance required to inhibit 1 mg of bovine pancreatic chymotrypsin [172,173].

Clemente et al. [66] reported that both BBI binding sites, displaying anti-trypsin and anti-chymotrypsin activities, might contribute to the antiproliferative effect on the colon cancer cells. Both major BBI isoinhibitors from pea (IBB1 having both trypsin and chymotrypsin inhibitory activities and IBBD2 with trypsin activity only) reduced the growth of human colorectal adenocarcinoma HT29 cells in vitro, in a dose-dependent manner. It was also shown that a chemically-modified analog, obtained upon reduction of disulfide bonds and subsequent alkylation of the sulfhydryl groups, lost its inhibitory and antiproliferative activities. This result was related to the serious disturbance of the inhibitor’s functional structure maintained by the disulfide bridge network. One of the finest evidences showing a direct association between inhibitory and antiproliferative activities of BBI was presented by Clemente et al. [165]. A recombinant BBI (named as rTI1B), representing a major BBI isoinhibitor from pea, was compared to its engineered analog having amino acid substitutions at the P1 positions in both inhibitory domains. rTI1B inhibited trypsin and chymotrypsin strongly and affected the growth of cancer HT29 cells, whereas its analog did not show any inhibitory and antiproliferative activities. Very recently, Olias et al. [120] showed that glycation of IBBD2 had a negative effect on trypsin inhibition and on HT29 cells growth. Interestingly, the second isoinhibitor IBB1 turned out to be less sensitive to glycation (heating in the presence of glucose) and maintained its strong inhibitory and anti-carcinogenic properties.

Table 3 summarizes the most important results, proving the ability of soybean-derived BBI, as well as its relatives from different sources, to prevent and suppress carcinogenic processes in the number of cancer cell lines and animal model systems. Based on promising reports, in April 1992, BBIC achieved Investigational New Drug status from the Food and Drug Administration (FDA), which opened the possibility to evaluate its anticarcinogenic potential in human clinical trials [22]. A 1-month phase IIa trial showed that 31% of patients (out of 32 patients) with oral leukoplakia achieved either partial or complete clinical response after treatment with BBIC. The inhibitor was administered as a single oral dose between 25 and 800 CIU per day [155]. BBIC was well-tolerated by patients and caused a reduction of the total lesion area. Later, BBIC was examined in a randomized, placebo-controlled phase IIb trial in 132 patients with oral leukoplakia [174]. It was administered as a single oral dose ranging from 200 to 1066 CIU/day. BBIC was non-toxic; however, no significant differences between BBIC treatment and placebo were reported. After 6 months of treatment, both placebo and BBIC caused a statistically considerable and highly comparable reduction of lesion area by 17.1% and 20.6% and clinical responses of 30% and 28%, respectively. It is likely that the unexpectedly high response of plant-derived placebo, containing various constituents with presumed cancer-preventive activity, could mask the clinical effect of BBIC. It was also implied that storage conditions (e.g., freezing) and time could affect the chemopreventive ability of BBIC. Furthermore, Malkowicz et al. [144] presented results of a phase I trial in nineteen male patients suffering from benign prostatic hyperplasia. BBIC was administered orally to patients for 6-months, two times a day, up to 800 CIU/day, and no toxicity was observed. The treatment caused a marked reduction of prostate-specific antigen (PSA) level in all patients, as well as a decrease in serum triglyceride level and prostate volume. The reported lack of dose-limiting toxicity of BBIC was further confirmed in two phase I randomized double-blind placebo-controlled trials performed in healthy male subjects. Two formulations of BBIC were administered at daily doses up to 2000 CIU, both as suspensions in orange juice [173]. Final BBI inhibitory activity in the original formulation, prepared as described elsewhere [172], was about 100 CIU/g, whereas in a novel, more concentrated extract, it was 562 CIU/g. The application of both formulations did not lead to any clinically relevant changes in hematological and biochemical parameters. Noteworthy, the bioavailability of BBI in the trial utilizing the novel formulation was about 40% to 43% of the bioavailability reached in the trial with the original formula.

Even though no toxicity of BBIC was observed yet, keeping in mind the digestive resistance of BBIs, it should be carefully examined if its activity is only local or systemic. It has been demonstrated that in humans, BBIs adsorbed in the GI tract from soymilk are detected in the urine shortly after ingestion in the unchanged form [175]. While in cannulated pigs, about 4–7% of ingested BBIs get to the distal ileum [135]. Billings et al. proved that BBI was internalized by intestinal epithelial cells [176]. Thus, after absorption, a large amount of BBI was distributed to tissues. Importantly, BBI is absorbed systemically in human subjects following oral administration of BBIC [173]. This means that they work both locally and systematically, thus indicating BBI’s bioavailability for chemoprevention of cancer not only in the GI tract but also in other organs. However, further investigation is needed to fully understand the BBIs mechanism of action and their distribution in serum and body organs.

#### Putative Mechanisms of Anticarcinogenic Activity

The question regarding the mechanism(s) by which BBIs exert their anticarcinogenic effects is still open and intriguing. Soybean BBI has been shown to present the capability to suppress the expression of various oncogenes (c-myc, c-fos) [22,177,178] and has been described as an inhibitor of the proteasome, which together with a small protein called ubiquitin, is responsible for the degradation of the majority of the proteins in eukaryotic cells [67]. An elevated proteasome concentration is observed in various tumor cells, which proliferate more rapidly than normal ones and require higher rates of protein turnover. Inhibition of proteasome results in an accumulation of proapoptotic and tumor suppressor proteins [179]. BBI inhibits, specifically, chymotrypsin-like (ChT-L) activity of 26S proteasome in vitro in MCF7 breast cancer cells [67]. Consequently, accumulation of ubiquitinated proteins and proteasome substrates (e.g., cell cycle regulators p27^kip1^ and p21^Cip1/WAF1^) has been reported, which, in turn, is associated with downregulation of cyclin D1 and E1, upregulation of mitogen-activated protein kinase phosphatase 1 (MKP-1), and suppression of phosphorylated extracellular signal-related kinases (ERK1/2) activity. These events result in cell-cycle arrest at the G_1_/S phase. The BBI-mediated inhibition of proteasomal activity, associated with a negative impact on osteosarcoma U2OS cells growth, was also reported by Saito et al. [158]. Consequently, the upregulation of a transmembrane protein connexin 43 (Cx43), considered as a tumor suppressor protein, was detected. It was shown that BBI stimulated the biosynthesis of Cx43 and suppressed its degradation. Notably, BBI did not affect Cx43 expression in non-tumorigenic cells. Similarly, BBI-induced expression of Cx43 has also been reported in mice with M5076 ovarian sarcoma [149,150], in human prostate cancer LNCaP cells, and in prostate adenocarcinomas in vivo model [147]. Besides soybean-derived BBI, another member of the family, the black-eyed pea derived BTCI, is able to inhibit proteasomal proteolytic activity. It forms a complex with horse erythrocytes 20S proteasome, as has been investigated by dynamic light scattering [60]. Based on immunofluorescent assays, BTCI enters MCF-7 cells in a time-dependent manner and is present inside them for 24 h. The colocalization of inhibitor and proteasome molecules has been reported. BTCI inhibits in vitro all active sites of the 20S proteasome. It is an even more potent inhibitor of trypsin-like (T-L) activity than well-known covalent peptide aldehyde MG132 and presents a similar inhibition to MG132 against ChT-L and caspase-like (C-L) activities. The rather unexpected inhibition of C-L activity (this site prefers mainly substrates and inhibitors with acidic amino acids in the P1 position) results most likely from conformational changes and subsequent steric hindrances, which might occur upon binding of BTCI to T-L and ChT-L sites. Another research confirmed that BTCI exerted a cytotoxic effect on MCF-7 [161] and highly invasive MDA-MB-231 [160] breast cancer cell lines, in a dose-dependent manner, without affecting normal mammary epithelial cells. Treatment of cells with BTCI caused a rapid increase of intracellular reactive oxygen species (ROS) level and loss of mitochondrial membrane potential following proteasome 20S inhibition [160]. Significant reduction of the cell viability and proliferation (arrest at S and G2/M phase) has been reported after 72 h incubation of cells with BTCI at the concentration of 200 µM [161]. Noteworthy, as determined by MTT assay, human normal breast MCF-10A cell viability is not affected under these conditions. Detailed analysis of MCF-7 cells treated with BTCI has revealed remarkable alteration in cell and nucleus morphology, plasma membrane fragmentation, cytoplasm disorganization, presence of double-membrane vesicles, increased DNA fragmentation, reduced mitochondrial membrane potential, increased mitochondrial size, as well as lysosome membrane permeabilization. All these observations suggest that BTCI is able to induce both the apoptosis and the lysosome membrane permeabilization processes.

Kaneko et al. [146] demonstrated the capability of soybean BBI to reinforce cytotoxicity induced by *α*-tocopheryl succinate (TOS) mitocan. As compared with individual BBI and TOS treatments, the combined application of these both agents resulted in a significant decrease in cell viability of prostate cancer cells (LNCaP) and undifferentiated prostate stem-like cells, which are more resistant to chemotherapy. It was shown that such a combination enhanced the induction of apoptosis via the caspase-9/caspase-3 pathway in the stem-like cells. The observed beneficial role of BBI was related to its ability to increase Cx43 expression, which, in turn, contributes to the differentiation of stem cells. It has also been proved that BBI stimulates DNA repair in p53 wild-type cells [180]. The inhibitor causes an increased internalization of epidermal growth factor receptor into the cytoplasm and stimulates its nuclear transport, which results in the activation of DNA-dependent protein kinase, a key player in DNA repair. BBI induces the suppression of growth of two cell lines—AGS (gastric adenocarcinoma cell line) and HT29 (colorectal adenocarcinoma)—which has been recently correlated with the inhibition of either secretion or activity of two matrix metalloproteinases—MMP-2 and MMP-9 [181]. Both enzymes are known to be involved in tumor invasion and metastasis. Moreover, treatment with BBI reduces the cellular secretion of vascular endothelial growth factor VEGF, a key player in tumor angiogenesis. The anti-angiogenic activity has also been reported in the case of the trypsin inhibitor extracted from the aqueous extract of *Cucumis melo* (melon) seeds, named TICMS [182]. TICMS decreases significantly the proliferation of human umbilical vein endothelial cells (HUVECs), inhibits in vitro cell motility and invasion, as well as reduces the secretion of MMP-2 and MMP-9 and VEGF, as indicated by the ELISA method. Molecular docking study has shown high tendencies for TICMS to bind the αVβ3 integrin within the cleft between the α and β subunits.

A different view on the role of soybean BBI in the fight against cancer was presented by Cruz-Huerta E. et al. [121]. They demonstrated that the major role of IBB1 (one of the BBI isoinhibitors) is to protect lunasin, a bioactive, soybean-derived peptide endowed with chemopreventive, antioxidant, and anti-inflammatory properties [183], from hydrolysis by gastric and pancreatic enzymes. If the ratio of lunasin:IBB1 was 1:2, more than 5% of lunasin remained intact upon hydrolysis conducted in vitro using a simulated gastrointestinal digestion model. For comparison, when chemically inactive, reduced IBBI1 was applied, and the residual concentration of lunasin was about 1.5%. It has been also shown, for the first time, that the mixture consisting of lunasin, IBB1, and related shorter peptides, obtained upon digestion, presents anti-proliferative effects on colon cancer cells (HT-29 and Caco-2) [183]. Hsieh et al. [153] examined in vivo effect of lunasin and BBI using the xenograft model of nude mice transplanted with human breast cancer MDA-MB-231 cells. As opposed to BBI, a significant reduction of cell proliferation and induction of apoptosis in the tumor cells was observed only in lunasin-treated mice. This result provides another evidence that the main function of BBI is to protect lunasin, the actual bioactive agent, from breakdown.

It should be emphasized that not all BBIs exert anti-cancer activity, even though they act as strong inhibitors of proteases. For example, trypsin-chymotrypsin inhibitors isolated from the seeds of the black gram (*Vigna mungo*) are not able to exert anti-proliferative effects on hepatoma (Hep G2) and breast cancer (MCF 7) cells [184].

### 5.2. Anti-Inflammatory and Immunomodulatory Properties

Serine proteases are known to be involved in tissue damage during inflammation. Among pro-inflammatory agents are neutrophil serine proteases, coagulation factors (plasmin, thrombin), granzymes, and some complement system enzymes, i.a. [185]. They participate in an inflammatory response by stimulating the production of pro-inflammatory mediators, including tumor necrosis factor TNF-α, interferon IFN-*γ*, interleukins, as well as chemokines, nitric oxide (NO), and prostaglandins [186,187]. They are involved in the maturation of pro-inflammatory cytokines zymogens [188], induce their pro-inflammatory activity [189], and influence their release from monocytes [190]. Thus, inhibition or modulation of the activity of serine proteases taking part in the production of inflammatory cytokines is deemed as a potential strategy in the treatment of inflammation diseases.

What makes BBI a good candidate for treating inflammatory diseases is its structure, making it resistant to proteolysis in the digestive system and ability to inhibit proteases produced from macrophages and mast cells during the inflammation, i.e., chymase [171], neutrophil elastase, and cathepsin G [191]. It also reduces the release of superoxide anion radicals from immunocytes [139] and suppresses the generation of active oxygen species in stimulated polymorphonuclear leukocytes [22,192] and in differentiated HL60 cells [193]. Noteworthy, BBI does not act as a free radical scavenger; however, the exact mechanism has not been established [193].

#### 5.2.1. Inflammatory Disorders of Gastrointestinal (GI) Tract

BBI’s anti-inflammatory properties are confirmed for inflammatory disorders of the gastrointestinal (GI) tract, such as inflammatory bowel disease (IBD), usually standing for ulcerative colitis (UC) or Crohn disease. In short, both manifest chronic inflammation of tissues in the GI tract and elevated activity of plasma proteases [194]. In the case of Crohn’s disease (dysregulation of type 1-helper cell, Th1), inflamed mucosal is present in the whole GI tract, including the mouth. UC (dysregulation of type 2-helper cell, Th2) usually means inflammation of colon, distant colon, and/or rectum [139,194]. In both, the disintegration of the intestine epithelium results in an uncontrolled permeation of antigens and microbes, including host bacteria, etc. [194]. Diet enriched with 0.5% BBIC leads to the suppression of mucosal inflammation in mice, reduced mortality by 15%, and suppressed histopathological inflammation criteria [139]. BBI’s anti-inflammatory properties have been confirmed in another experiment [195] in which mice with induced colitis were fed with pea seed extract, albumin fraction from pea seed extract, or pure BBI. Similar to previous research [139], the anti-inflammatory effect was observed, and the presence of inflammation markers, as well as the symptoms, was diminished. Interestingly, also, the lower release of proinflammatory cytokines and Toll-like receptors has been demonstrated [195]. Promising results obtained for mice with IBD have encouraged to examine BBIC treatment of IBD in humans [196]. Clinical trials have shown the amelioration of the IBD patients’ condition and also the reemission of UC after 12 weeks of BBIC therapy. Neither toxicity nor adverse side effects are observed [196]. Although the obtained results have undoubtedly demonstrated the superiority of BBIC over placebo, they have not achieved statistical significance, and a more extensive trial is required. Another fermented soy germ extract (reach in BBIs and isoflavones) [197] has reduced colitis in a rat model, and it is BBI that has contributed to the silencing of protease-activated receptor 2 expression and reduced fecal proteolytic activity.

#### 5.2.2. Experimental Autoimmune Encephalomyelitis

Encouraging results of BBI application in the treatment of inflammations, as well as its good tolerance in clinical trials, have induced further research on its therapeutic potential. Experimental autoimmune encephalomyelitis (EAE), the animal model of multiple sclerosis (MS) [198], is directly caused by the production of inflammatory cytokines, and it is connected with elevated protease activity, which leads to demyelination. Thus, BBI is thought to be an excellent candidate to treat also MS [198,199]. Indeed, the oral administration of BBIC has brought good results in the treatment of EAE rats, diminishing the symptoms, and delaying the onset [199]. It has also been demonstrated that the lymph node cell proliferation decreases ex vivo due to the inhibition of myelin basic protein (MBP)-specific T-cell activation [199]. BBIC efficacy in the treatment of MS animal model has resulted from the increased production of IL-10, an EAE-suppressive, anti-inflammatory cytokine [12,200]. It has been proved that BBI induces the release of IL-10 in both peripheral and infiltrating immune cells and increases its production in CD4^+^ T cells [200]. Treatment of EAE mice with BBI has resulted in elevated IL-10 production and, thus, improved disease-parameters, i.e., onset, severity, weight loss, inflammation, and demyelination [12]. The effectiveness of BBI treatment is confirmed also in human cells using peripheral blood mononuclear cells (PBMCs) from healthy donors and MS-treated donors. In both cases, the presence of BBI has led to induced IL-10 release, which indicates its potential to be used in MS therapy [201].

#### 5.2.3. Experimental Autoimmune Neuritis

Similarly, promising results [202] have been obtained in the treatment of mice with experimental autoimmune neuritis (EAN), the animal model of Guillain–Barre syndrome (GBS), and other autoimmune diseases. GBS is a complex autoimmune disease of the peripheral nervous system, leading to acute flaccid paralysis. It may progress so rapidly that the patient becomes ventilator-dependent within days. It is usually caused by acute inflammatory demyelinating polyneuropathy, although various clinical variants of GBS are reported [203]. EAN is associated with segmental demyelination, axonal injury, and activated T cells and macrophages infiltration into the peripheral nervous system, which is related to elevated production of pro-inflammatory cytokines [204]. Daily BBIC administration to EAN mice reduces demyelination in the peripheral nervous system and inflammation, affecting the amelioration of its symptoms, including paralysis [202]. Interestingly, in rats treated with BBIC, the shift of pro-inflammatory macrophage state M1 to anti-inflammatory M2 has been observed, which is reflected in the decreased production of pro-inflammatory cytokines (e.g., TNF-*α*, IFN-*γ*), and an increased release of these is considered as an anti-inflammatory (e.g., IL-10, IL-4). These results have shed new light on the treatment of GBS and other autoimmune diseases in humans. Recent in vivo investigation [186], using lipopolysaccharide inflammation-induced mouse model, has confirmed BBI’s anti-inflammatory properties, leading to reduced TNF-*α*, IFN-*γ* serum levels. Four variants of potential therapeutics have been tested: BBI alone, soybean-derived anti-inflammatory agent genistein alone, BBI and genistein combination, and the BBI-genistein conjugate. The best results are observed for combined therapy using BBI together with genistin.

#### 5.2.4. Alzheimer’s Disease

It has been suggested that soybean BBI could be effective in the inhibition of Alzheimer’s disease. Akbari et al. [205] studied the effect of BBI on in vitro model of Alzheimer’s disorder in which accumulation of amyloid β (Aβ) in PC12 cells was stimulated with HgCl_2_. It was shown that BBI inhibited the accumulation of Aβ by inducing autophagy and decreased apoptosis, which is regarded as a key event in neurodegenerative diseases. The expression of several genes associated with autophagy (*Atg5*, *Beclin1*, and *Bnip3*) and apoptosis (*Bax* and *Bcl2*) was changed under BBI treatment.

#### 5.2.5. Anti-Inflammatory Properties of Animal-Derived Bowman-Birk Like Inhibitors

**Figure 1:**
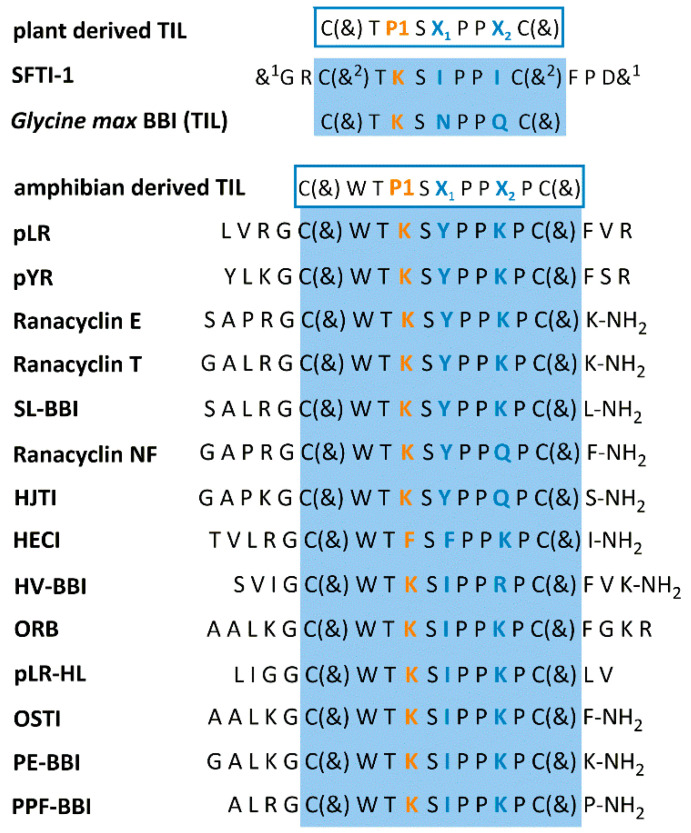
The comparison of trypsin inhibitory loops (TILs) of amphibian-derived BBLTIs and representatives of plant-derived BBIs (*Glycine max* BBI and SFTI-1). P1 position is marked in orange, and variable X_1_ and X_2_ positions are marked in blue. The conserved disulfide loop of amphibian BBLTIs is highlighted in blue.

Some of the amphibian skin-derived BBLTIs are reported to have anti-inflammatory properties. An example is peptide leucine-arginine (pLR) from the Northern Leopard frog (*Lithobates pipiens*, formerly *Rana pipiens*) [206]. The primary structure of this peptide is LVRGC(&)WTKSYPPKPC(&)FVR, and its name, pLR, comes from its *N*- and *C*-terminal residues. pLR is described as the first and the most potent noncytolytic histamine-liberating peptide of natural origin, exhibiting a 2-fold higher activity in comparison to melittin—reported as one of the strongest histamine-releasing peptides. Additionally, it inhibits granulopoiesis, but unlike other inhibitors, its activity is directed only against myeloid progenitor cells, and no effect is observed in the case of mature neutrophils [206]. Further investigation has proved that it is a potent inhibitor of tryptase involved in allergic asthma (kind of bronchial inflammation), among others [207]. Experiments on a murine asthma model have proved that pLR decreases the acute asthma-like phenotype and airway remodeling and chronic airway inflammation symptoms [208]. Tryptase inhibition has been examined in vivo in ovalbumin (OVA)-immunized mice. It turns out that the proteolytic activity in the bronchoalveolar lavage (BAL) fluid of animals treated with pLR is decreased up to 60% (conc. 100 nM) [208]. Interestingly, tryptase active site is not accessible for macromolecular ligands, such as BBI, due to its tetramer composition with active site located in the center. Shortly after pLR, another peptide of amphibian skin origin was discovered [209], namely peptide tyrosine-arginine (pYR) from dusky gopher frog *Lithobates capito* (formerly *Rana sevosa*) skin, which shares 77.8% homology with pLR (Figure 1). Similar to pLR, pYR also exhibits immunomodulatory potency. Granulopoiesis inhibition is observed at the same level as for pLR, and it is the most significant in the case of analogs with a carboxyl group in the *C*-terminus. The non-amidated pYR analog is also the most potent (among three analyzed analogs, amidated and non-amidated in the *C*-terminus, and the disulfide-bridged loop fragment) inhibitor of progenitor cells. Similar to pLR, the immunomodulatory activity of pYR is stage-specific and does not lead to the apoptosis of mature neutrophils [209].

#### 5.2.6. Putative Mechanisms of Anti-Inflammatory Activity

Although BBI’s efficacy as an anti-inflammatory agent is unquestionable, its mechanism of action is still not understood. There are some presumptions trying to explain its mode of action. One assumes its inhibitory potency towards proteases is released excessively by the immune cells during the inflammation process (e.g., elastase, chymase, cathepsin G), contributing also to reduced proinflammatory cytokines release [139,202]. The other relates BBI anti-inflammatory potency to blocking the production of superoxide anion radicals by immunocytes, thus mitigating oxidative stress (e.g., muscle atrophy and weakness) [210]. Immunoprotective abilities of BBIC are also attributed to its ability to restrict proliferation and transformation of cells through the modulation of the functioning of c-myc and c-fos oncogenes [202].

### 5.3. Antimicrobial Activity

#### 5.3.1. Antiviral Activity

Reports regarding antimicrobial activities of BBIs are scarce. However, BBI is proved to display antiviral activity toward bovine herpes virus-1 [211], herpes simplex virus type 2 (HSV-2) [212], and HIV [213,214]. Interestingly, HSV-2 infection is considered to facilitate HIV-1 sexual transmission.

HSV-2 infection of human cervical epithelial cells (End1/E6E7) is inhibited in vitro at both DNA and protein levels [212]. The therapeutic effect exerted by BBI is observed at different conditions (before and after HSV-2 infections) and, notably, is not associated with cell cytotoxicity. BBI treatment decreases the expression of various HSV-2 genes in cells, which are recognized as key players in the virus replication. On the other hand, the expression of interferons (IFN-*α*, IFN-*λ*1, and IFN-*λ*2/3) and several antiviral interferon-stimulated genes (ISGs) is enhanced, which, in turn, results in the activation of the Janus kinase/signal transducers and activators of transcription (JAK/STAT) signaling pathway. Moreover, BBI treatment of cells partially inhibits the cellular ubiquitin-proteasome system and suppresses HSV-2-induced activation of NF-κB and p38 MAPK signaling pathway. Finally, the upregulated expression of tight junction proteins, which help to maintain the integral epithelial barrier for the virus, has been reported.

Various BBIs are presented as potent in vitro inhibitors of HIV reverse transcriptase, for example, inhibitors from small glossy black soybeans (IC_50_ of about 0.16 μM [215]), seeds of Faba bean (IC_50_ of about 0.76 μM [83]), seeds of Hokkaido large black soybeans (IC_50_ of about 38 μM [152]), and broad beans (58% inhibition at 49 μM) [216]. Ma et al. provided evidence that soybean BBI was able to inhibit HIV infection of peripheral blood monocyte-derived macrophages without cytotoxicity [213]. The most efficient inhibition was achieved when cells were subjected to simultaneous BBI treatment and HIV infection. Noteworthy, the treatment with inhibitor, both before and after infection, resulted in significant inhibition. BBI selectively induced the expression of IFN-*β*, as well as multiple ISGs and several key HIV restriction factors, which could inhibit viral replication at different stages. Further research has indicated that BBI is able to inhibit infection of macrophages at the entry-level [214]. It has been shown that BBI downregulates the expression of primary receptor CD4 (as much as 80%) and, less significantly, chemokine receptor type 5 (CCR5). It is known that the binding of viral envelope glycoprotein gp120 to both receptors precedes the fusion of the viral and cell membranes. Moreover, BBI induces the production of the CC chemokines in macrophages. These chemokines act as ligands for CCR5 and could inhibit the entry of HIV into cells.

#### 5.3.2. Antifungal Activity

Several BBIs have been reported to possess antifungal activity [77,216,217,218]. A trypsin inhibitor from wheat kernel has presented a wide spectrum in vitro antifungal activities against different fungi, with IC_50_ values ranging from 111.7 to above 500 μg/ml [218]. It is possible that the inhibitor blocks proteolytic activation of the chitin synthase zymogen, which is involved in the chitin biosynthetic process during fungal cell wall development. An inhibitor isolated from *D. biflorus* seeds has reduced the growth of phytopathogenic fungi *Alternaria alternata* (minimum inhibitory concentration, MIC 0.4 μg/ml), *Fusarium oxysporum* (MIC 0.6 μg/ml), and *Aspergillus niger* (MIC 1.2 μg/ml) [77]. There are many examples showing that the plant defense system is subverted by pathogen inhibitors targeting host proteases. In contrast, only a few studies describe the opposite situation in which pathogens proteases interact with the host inhibitors [219]. Rice blast, caused by the fungal pathogen *Magnaporthe oryzae,* is one of the most destructive rice diseases. The fungal avirulence effector AvrPiz-t is released into the rice cell during infection to suppress the host immune system. The effector is recognized by the cognate host resistance protein Piz-t; however, these two proteins do not interact directly. Zhang et al. [220] showed that the rice (*Oryza sativa*) BBI protein, named APIP4 (AvrPiz-t interacting protein 4), was involved in this interaction and played a positive role in rice immunity. The fungal AvrPiz-t directly targets APIP4 and reduces its inhibitor activity in vivo and in vitro. On the other hand, the rice resistance protein Piz-t boosts APIP4 expression, its accumulation, and trypsin inhibitory activity. APIP4 knockout plants display enhanced susceptibility to the fungal pathogen, whereas the overexpression of APIP4 in transgenic rice positively regulates resistance to the pathogens.

#### 5.3.3. Antibacterial Activity

Regarding antibacterial action, BBI isolated from the seeds of leguminous plant *Luetzelburgia Auriculata* (LzaBBI) has exerted in vitro activity against human pathogenic Gram-positive bacteria *Staphylococcus aureus* with MIC and minimum bactericidal concentration (MBC) values of 23.1 × 10^−4^ and 92.5 × 10^−4^ μM, respectively [74]. As it is shown, LzaBBI disrupts the bacterial membrane’s integrity and increases the intracellular generation of ROS, eventually leading to bacterial death.

### 5.4. Insecticidal Activity

As some BBIs are wound- or defense-inducible [221], one of their highly plausible functions in plants is to provide protection against insects, which utilize proteases (mostly trypsin- and chymotrypsin-like proteases) to digest ingested proteins. Inhibition of the proteolytic activity of these enzymes impairs digestion and results in a subsequent deficiency of essential amino acids. This, in turn, negatively affects larval growth and development, reduces fecundity and fertility of the adult organisms, and eventually may cause their death [222]. Moreover, such an inhibition triggers massive overproduction of proteases by insects, which seriously affects the availability of amino acids required for the production of other essential proteins. Prasad et al. [223] showed that two Bowman-Birk inhibitors isolated from the seeds of a red gram (*Cajanus cajan,* RgPI) [75] and black gram (*Vigna mungo*, BgPI) [78] displayed inhibitory potencies against various lepidopteran insects. Both inhibitors showed remarkable in vitro inhibitory activity against trypsin-like proteinases found in the midgut extract prepared from *Achaea Janata*, a devastating pest of the castor plant, compared to that of soybean BBI. Conversely, the inhibition of midgut trypsin-like proteinases from other insects, such as *Helicoverpa armigera*, *Papilio demoleus,* and *Amsacta albistriga,* was either marginal (RgPI) or moderate (BgPI, BBI). Feeding of *A. janata* larvae on leaves coated with RgPI caused significant, dose-dependent, linear reduction in larval body weight and survival rate. For example, after 6 days of feeding on the leaves covered with 4 μg of RgPI per cm^2^ of leaf area, about 50% retardation in larvae body weight was observed. The survival rate was found to be 52%, as compared to the control. In contrast to RgPI, BgPI showed a significant reduction in body weight of *Spodoptera litura* larvae after feeding with an inhibitor-supplemented artificial diet for 7 days.

Recently, the BBI gene has been cloned from the immature seeds of *Rhynchosia sublobata,* and a recombinant inhibitor, named rRsBBI1, has been overexpressed in *Escherichia coli* [129]. rRsBBI1 has shown significant in vitro inhibition of the gut trypsin-like proteases of *A. janata* larvae (but not *H. armigera*), induces its growth retardation, and increases the mortality rate. At the end of the 11th day of feeding on leaves coated with rRsBBI1, the bodyweight of the *A. janata* larvae decreases significantly, up to 84% of the control weight. The significant inhibitory activity of pure RsBBI against gut trypsin-like protease (IC_50_ = 24 ng) was further confirmed by Mohanraj et al. [55]. Another inhibitor displaying insecticidal activity against *A. janata* larvae has been extracted from the seeds of the non-host plant *Cajanus cajan* and named C11PI [76]. The feeding on leaves coated with C11PI (2–8 μg/cm^2^) has significantly increased mortality, reduced larval (55–71% of control) and pupal (33–55%) body weights, delayed transition from larva to pupae, and led to the formation of abnormal intermediates. Recently, a peanut Bowman-Birk inhibitor (PnBBI) isolated from the seeds of interspecific hybrid peanut variety has been shown to display insecticidal potential against *H. armigera*, which is one of the most devastating pests in agriculture [56]. Under in vitro conditions, PnBBI has presented strong inhibitory activity against larvae midgut trypsin-like proteases, whereas in vivo feeding assays have shown a dose-dependent reduction of larval body mass. The highest mass decline (42% of control) has been observed upon 6 days of feeding with a test diet supplemented with 0.005% PnBBI. Interestingly, the inhibitor has influenced the expression of trypsin-like proteases. Based on one and two-dimensional zymography studies, several enzymes are depleted upon PnBBI feeding.

Dantzger et al. [81] showed that protease inhibitor from *Clitoria fairchildiana* seeds (CFPI) exhibited significant in vitro inhibitory activity against larval midgut trypsin-like enzymes from *Anagasta kuehniella* (enzymatic activity lowered by 76%), *Diatraea saccharalis* (59%), and *Heliothis virescens* (49%). Feeding the larvae of *A. kuehniella* (the Mediterranean flour moth) with an artificial diet containing 1% of CFPI reduced its weight by about 40% and affected the survival rate, which was reduced by 27.5%. The larvae showed stunted growth and a prolonged period of development in an adult. The chronic ingestion (since neonate larvae until adult stage) of CFPI caused lower efficiency of the conversion of ingested food (ECI) and efficiency of conversion of digested food (ECD) when compared with control-fed larvae.

Insecticidal activities of BBIs against various aphids—*Acyrthosiphon pisum* [84] and *Macrosiphum euphorbiae* [224]—have also been described. Azzouz et al. [224] examined an effect of soybean BBI on potato aphids *M. euphorbiae* and its endoparasitoid *Aphelinus abdominalis*. Feeding with an artificial diet supplemented with this inhibitor did not affect the nymphal viability of *M. euphorbiae* but significantly altered adult demographic parameters, e.g., reduction of daily fecundity was reported. On the contrary, cysteine protease inhibitor oryzacystatin I substantially reduced the nymphal survival of *M. euphorbiae* and prevented aphids from reproducing. Parasitoids developed in aphids fed with a diet containing either soybean BBI or oryzacystatin showed a fitness impairment, even though only the first compound was detected in parasitoid larvae. BBIs display also insecticidal activity against coleoptera species, such as pest devastating coffee crops (*Hypothenemus hampei* [225]), cotton (*Anthonomus grandis* [226], *H. armigera* [77]), maize (*Prostephanus truncatus* [227]), and stored grain (*Tribolium castaneum* [228]).

### 5.5. Other Putative Functions

BTCI is shown to be involved in guanylin-induced natriuresis and, as the first BBI, is presented to play a stimulating effect on renal function in rats, which is likely associated with its inhibitory activity [229]. It is suggested that BTCI protects chymotrypsin-driven degradation of guanylin, a small natriuretic peptide (15 amino acids) that regulates electrolyte and water transport in intestinal and renal epithelia. If analyzed alone, guanylin (0.2 µM) does not induce changes in kidney function. However, its pretreatment with BTCI (0.3–3.0 µMleads to changes in ex vivo renal function manifested by increase in urine flow, fractional excretion of Na^+^ (for 0.3 µM BTCI, change of %ENa^+^ from 22.7 ± 0.68% to 30.0 ± 2.84%, *p* < 0.05, after 120 min) and K^+^ (for 1.0 µM BTCI, change of %EK^+^ from 43.2 ± 3.19% to 65.7 ± 9.67%, *p* < 0.05, after 90 min), glomerular filtration rate (for 3.0 µM BTCI from 0.96 ± 0.02 mL g^−1^/min to 1.28 ± 0.02 mL g^−1^/min, *p* < 0.05, after 60 min), perfusion pressure (for 3.0 µM BTCI, increase from 110.1 ± 1.66 mmHg to 151.8 ± 2.08 mmHg, *p* < 0.05, after 120 min), and osmolar clearance (for 3.0 µM BTCI, from 0.13 ± 0.02 mL g^−1^/min to 0.23 ± 0.01 mL g^−1^/min, *p* < 0.05, 60 min). BTCI, either free or in non-covalent complex with endogenous biologically active peptide bradykinin, also affects cardiovascular functions in rats after intravenous administration [230]. Both constituents of the complex retain their original functions. BTCI inhibits trypsin and chymotrypsin, while bradykinin displays effects on smooth muscle. The presence of an inhibitor promotes a decrease of vascular resistance and hypotension and improves renal and aortic vasodilation induced by bradykinin. The authors hypothesized that BTCI might function as a carrier for bradykinin in the blood in order to protect it from proteases. Antihypertensive and vasodilator effects of BTCI and its two related disulfide-bridged nonapeptides, reflecting its trypsin and chymotrypsin binding loops, are also demonstrated in the further study [231]. BTCI and both synthetic peptides promote a decrease of systolic (SBP) and diastolic blood pressure (DBP) and renal and aortic vasodilation in normotensive (Wistar-WR) and spontaneously hypertensive rats (SHR) after gavage administration. In the case of Wistar-WR, administration of BTCI (at dose 30.0 mg·kg^−1^) decreases the value of SBP from 140.1 ± 1.3 to 102.0 ± 6.3 mmHg, while DBP from 91.5 ± 3.0 to 51.7 ± 3.5 mmHg. Regarding SHR, SBP is reduced from 175.4 ± 2.5 to 113.3 ± 4.8 mmHg, whereas DBP from 121.3 ± 2.7 to 59.9 ± 4.1 mmHg. On the other hand, renal vascular conductance (72 ± 15.3% in WR and 78 ± 10.1% in SHR) and aortic vascular conductance (50.1 ± 6.5% in WR and in 80.3 ± 11.5% SHR) are increased. Additionally, BTCI and the related peptides induce coronary vasodilation in isolated hearts, which is likely mediated by the endothelial nitric oxide synthase/nitric oxide (eNOS/NO) pathway. It is speculated that the blood pressure-lowering effect is associated with the inhibition of angiotensin-converting enzyme with IC_50_ value 54.6 µM [231].

### 5.6. Antinutritional Activity

Despite all the above-mentioned BBIs benefits, we should be aware of some possible deleterious side effects that might result from the intake of these inhibitors. Due to their high stability in the GI tract, BBIs alongside tannins and phytic acid are considered antinutritional factors [232]. They may reduce the activity of pivotal enzymes within the gastrointestinal tract of animals, leading to lower digestion and adsorption of dietary proteins. This may result in the inhibition of organism growth and pancreatic disorders, such as hypertrophy and hyperplasia. Therefore, in recent years, various techniques have been developed to reduce the contents and activity of inhibitors in soybean foods [233], especially soy milk, which is a common substitute for animal milk. BBI concentration in commercial soy milk is 11.83 ± 5.55 mg/g of protein and 27.65 ± 15.39 mg/100 mL of milk [234]. Besides the most commonly used thermal techniques, a number of alternative non-thermal approaches have been introduced, including high hydrostatic pressure, instantaneous controlled pressure, ultrasound, extrusion, germination, and fermentation [233]. Chen et al. reported that two major components of tea polyphenols—epigallocatechin gallate and epigallocatechin—effectively reduced the inhibitory activity of BBI [235]. The binding between both phenols and BBI, stabilized by hydrophobic interactions and hydrogen bonds, markedly changed the conformation of the protein. Li et al. [236] presented that dielectric-barrier discharge plasma treatment might be regarded as a novel approach to inactivate soybean trypsin inhibitor.

### 5.7. SFTI—An Exceptional Member of BBIs

SFTI-1 is a disulfide-bridged 14 amino acid backbone-cyclized natural peptide found in the seeds of sunflower (*Helianthus annuus*). Its exposed binding loop is almost identical to that found in other bigger BBIs [51]. As was shown by Mylne et al. [50], SFTI-1 was excised and simultaneously macrocyclized, likely by asparaginyl endopeptidase(s), from its linear albumin precursor protein called preproalbumin with SFTI-1 (PawS1). Due to its small size and compact and rigid spatial structure, SFTI-1 has been considered as an extremely attractive starting compound to design novel, potent, and highly selective inhibitors, blocking various proteases (see Table 2). SFTI-1 sequence has also been adapted to design analogs, for example, by applying molecular grafting approach, with various biological properties, among the others: peptides with anti-angiogenic activity [237], selective ligands for melanocortin receptor (MC1R) [238], the antagonist of bradykinin B1 receptor [239], or grafted peptides able to identify and neutralize selected autoantibodies associated with rheumatoid arthritis [240]. SFTI-1 has been reviewed very thoroughly in recent years, either among other cyclic, disulfide-bridged peptides [241,242] or alone [243], including the most recent, comprehensive review by Craik group [117]. In this work, the selected data regarding the inhibitory activity of SFTI-1 and its synthetic analogs are collected in Table 2.

### 5.8. Amphibian-Derived Bowman-Birk-Like Trypsin Inhibitors (BBLTIs)

In recent years, the interest in amphibian-derived BBLTIs is growing rapidly, which is reflected in an increasing number of publications reporting newly isolated members of this group [30,31,32]. Recently, a novel I99 family in the MEROPS database dedicated to such Bowman-Birk-like inhibitors has been established [2]. Amphibian-derived BBLTIs were isolated from frogs’ skin secretions. Names of specific species, together with inhibitors’ sequences and their various activities, are summarized in Table 4. As it was mentioned before, these compounds are endowed with a characteristic solvent-exposed trypsin-inhibitory loop (TIL), which is highly similar to that found in plant BBIs, see Figure 1. TIL of BBLTIs comprises 11 amino acid residues, and its general formula is CWTP1SX_1_PPX_2_PC, where X_1_ and X_2_ are variable, occupied usually by Tyr, Phe, or Ile in the case of X_1_ and Lys, Gln, or Arg at X_2_ position. In the P1 position, there is Lys in almost all representatives of BBLTIs. They are usually composed of 17 or 18 amino acid residues, with the exception of ORB and PPF-BBI, which are 20 and 16 residues long, respectively. Structures of amphibian-derived BLTIs are presented in Figure 1.

First examples of amphibian-derived BBIs are peptide leucine-arginine (pLR) [206] and peptide tyrosine-arginine (pYR) [209]. They both attract attention due to their stage-specific immunomodulatory activity, described here in the Section 5.2.5. pLR is also proved to be a potent inhibitor of trypsin, and its inhibitory potency towards tryptase is declared; however, the *K_i_* value for the latter is not provided [206]. It maintains inhibitory activity towards trypsin, even after *N*- and *C*-terminal truncation [208] (Table 4). Other BBLTIs are two 17 amino acids ranacycylins E and T [244], sharing high sequence homology to pLR (Figure 1). Unlike pLR, they are amidated at the *C*-terminus. Both ranacyclins, despite high sequence similarity, have demonstrated different activity against various bacteria strains with the superiority of ranacyclin T [244] (Table 4). For comparison, pLR has demonstrated activity only towards Gram-positive bacteria. Regarding antifungal activity, ranacyclin T is the most while pLR the least active against tested species. Noteworthy, ranacyclin E and pLR exert high hemolytic activity after 30 min and 60 min incubation, respectively. Previously, Salmon et al. [206] reported no hemolytic activity for pLR; presumably, it was caused by not enough incubation time.

Initially, pLR and pYR were not assigned to BBIs as their resemblance to this group was not so evident. However, in 2007, Li et al. [31] reported another peptide isolated from frog’s skin secretion, named ORB, suggesting its relationship to the BBI family, focusing on the reactive loop similarity and the identical fragment of Thr-Lys-Ser-Ile-Pro-Pro (P2-P4′) present in both ORB and plant-derived SFTI-1. Further evidence was delivered by Rothemund et al. [208], who compared the solution structures of binding loops of SFTI-1 and pLR with the crystal structure of reactive loop of soybean, classical BBI. Even though their disulfide-bridged loops differed in size, the close similarity of the three structures was evident [208]. The relationship of pLR to SFTI-1 was further demonstrated in functional analyses of certain pLR modifications, e.g., backbone cyclization, replacement of crucial residues by Ala. The examination of inhibitory potency of such analogs revealed that they acted similarly to SFTI-1 (i.e., increased/decreased inhibitory potency) [208]. Similarly, as SFTI-1, animal-derived BBLTIs have evolved independently from other plant BBIs [245,246].

The inhibitory activity of ORB towards trypsin is rather moderate when compared to other naturally occurring trypsin inhibitors; however, its truncated analog, yielding a disulfide-bridged hendecapeptide loop (named ORB-C), has exhibited about 430-fold increased inhibitory potency towards trypsin (Table 4). Various truncated ORB analogs are tested regarding their inhibitory and antimicrobial properties (Table 4). An intriguing combination of these two activities is observed for ORB and its four analogs, whereas six other peptides show improved inhibitory potency; however, lost antimicrobial activity (Table 4). Hu et al. [247] suggested that the bactericidal activity of ORB-1 (*E. coli* MIC 2.34 µg/ml; *S. aureus* 1.76 µg/ml; *Bacillus subtilis* 2.34 µg/ml, and fungi *Candida albicans* 4.69 µg/ml) was associated with its ability to form membrane-spanning channels. Since monomeric ORB-1 peptides are too short to span the bacterial membrane on their own, they form dimers in which the *C*-terminal carboxyl group of one ORB-1 monomer is associated with the *N*-terminal amine group of the second peptide by electrostatic attraction. Such clusters of dimers may disturb an integration of the bacterial membrane. This coincides with the inactivity of *C*-terminally amidated ORB-1 analogs, which are unable to form dimers. Unfortunately, the bactericidal activity of ORB-1 was not confirmed in our recent work [248]. No antimicrobial activity was observed for ORB-1 at a concentration range up to 250 µg/mL.

Strong antitrypsin activity is also presented by another BBLTI extracted from the skin of Chinese bamboo odorous frog *Huia versabilis*, named HV-BBI (*K*_i_ 18.8 nM [249], 3 nM [250]). HV-BBI is the *C*-terminally amidated peptide, which, similarly to pLR and pYR, is composed of 18 amino acid residues, and, like ORB, it contains Thr-Lys-Ser-Ile-Pro-Pro motif. The crystal structure of its truncated analog HV-BBI(3-18) with bovine *β*-trypsin (ODB: 4U2W) has proved that the formation of the peptide-enzyme complex is mostly stabilized by direct interaction between inhibitor’s Lys residue and the enzyme’s S1 pocket [250]. Antimicrobial screening [251] has revealed that HV-BBI and its truncated analogs display only minor activity in liquid Davis minimal mineral medium, whereas they are inactive when assayed in Mueller Hinton Broth. Replacement of Lys in P1 of HV-BBI with Arg has resulted in a decrease of inhibitory activity against trypsin, and both P1-Lys and P1-Arg analogs are not active against chymotrypsin [249]. In general, the truncated HV-BBI analogs (at both *N*- and *C*-terminus) display slightly lower inhibitory activity toward trypsin and plasmin in comparison to the native peptide [248,251]. Similarly, in the case of pLR-HL [252] and Hejiang trypsin inhibitor (HJTI) [253], the removal of *N*- and *C*-terminal fragments (yielding disulfide loops exclusively) leads to a 9- and 220-fold decrease of inhibitory activity against trypsin. Surprisingly, one of the shortest analogs, HV-BBI(4–16) deprived of *N*-terminal tripeptide and *C*-terminal dipeptide, is the strongest matriptase-1 inhibitor with *K*_i_ 8 nM [248]. Interestingly, such significant inhibitory activity is observed only for this terminally amidated peptide, whereas its disulfide-bridged analogs having either free *C*-terminal carboxyl group or continuous backbone are about 67- and 59-fold weaker, respectively.

The majority of naturally occurring amphibian BBIs show significant inhibitory activity towards trypsin; however, a subtle modification (i.e., replacement of P1 Lys with Phe) within their primary structure has resulted in novel inhibitors of chymotrypsin, as in the case of pLR-HL [252], OSTI [254], and PPF-BBI [32]. Up to date, HECI has been the only amphibian-derived BBLTI of chymotrypsin and the chymotrypsin-like activity of the human 20S proteasome [167]. Both HECI and its analog modified in the P1 position [Lys^9^]HECI have an antiproliferative effect on cancer cells (PC-3, H157, and MCF-7) at 1 mM. Nevertheless, HECI exhibits more potent anticancer activity and is able to inhibit the growth of PC-3 and H157 at 100 μM [167]. The reported differences in toxicity between both peptides might result from higher hydrophobicity of HECI due to the presence of hydrophobic motif -Trp-Thr-Phe-Ser-Phe-Pro-Pro-, which contributes to better membrane interaction. Besides, CD spectra in a membrane-mimic environment have demonstrated a 2-fold greater helix content in HECI as compared to its Lys-containing analog. Importantly, both peptides have demonstrated low cytotoxic and hemolytic activities [167]. Similarly, the analog of recently isolated SL-BBI [169], F-SL with Phe in the P1 position, has presented inhibitory potency towards chymotrypsin and proteasome and exerted antiproliferative effect in a series of cancer cell lines, including non-small cell lungs (H157, H460, H838, and H23), prostate (PC-3), and breast (MCF-7) [169].

Soon after, another BBLTI with anticancer properties was described [168]. PE-BBI, which is characterized by moderate trypsin inhibitory potency and lack of antibacterial activity, has attracted attention due to its strong myotropic activity on isolated rat bladder (EC_50_ 14.25 nM) and rat-tail artery smooth muscle relaxation (EC_50_ 8.79 nM). PE-BBI has revealed cytotoxicity towards human colorectal cancer cell lines (DLD-1, DKS8, HCT116, and HKE3), while no toxicity against human colon endothelial cell line. Authors speculated that anticancer activity of PE-BBI is associated with its higher positive charge, as compared to other non-active amphibian-derived peptides resulting from the presence of additional Lys and amidated *C*-terminus. Similarly, Miao et al. [32] demonstrated a chimeric peptide, named Tat-loop, composed of *N*-terminal Tat_48–56_ cell-penetrating peptide and a *C*-terminal binding loop derived from Bowman-Birk-type inhibitor (PPF-BBI) isolated from the skin secretion of the Fukien gold-striped pond frog, *Pelophlax plancyi fukienesis*. As opposed to PPF-BBI, Tat-loop with increased positive charge density has demonstrated higher antiproliferative activity against lung cancer cell lines H460 and H157, as well as significant antifungal potency towards *C. albicans.*

The relationship between the net charge and activity has also been observed in the case of very recently isolated ranacyclin NF (RNF) (from East Asian frog, *Pelophylax nigromaculatus*) [30] and SL-BBI (broad-folded frog *Sylvirana latouchii*) [169]. Similar to HJTI, RNF and SL-BBI (Figure 1) are moderate trypsin inhibitors. However, despite high sequence resemblance, RNF is 2-fold weaker than SL-BBI and almost 4-fold weaker than ranacyclin T. This difference seems to be correlated with a net charge, which is +5 in the case of ranacyclin T and +3 for SL-BBI, RNF, and HJTI. Regarding the last one, the replacement of Gln14 and Ser17 with l-lysines has increased net positive charge from +3 to +5 and slightly improved inhibitory potency towards trypsin and antimicrobial activity against *E. coli* [253]. Similarly, SL-BBI analog, K-SL, with a higher positive charge, has exhibited stronger antimicrobial potency and anti-trypsin activity. Moreover, the increased *α*-helical content has contributed to better antimicrobial abilities of K-SL [169]. It has also been observed for RNF and its analogs that these with limited possibility to *α*-helix or *β*-sheet formation are weaker trypsin inhibitors, similarly to peptides with *C*-terminal carboxyl group [30]. Interestingly, only RNF has demonstrated weak antimicrobial activity against *S. aureus,* while its analog with the *C*-terminal carboxyl group is inactive [30]. Interestingly, antibiotic gentamicin, when applied together with RNF, has shown a lower MIC value against methicillin-resistant *S. aureus*.

A molecular docking study demonstrated that PE-BBI has a high affinity to the active sites of several kallikreins (KLK4, KLK6, KLK8, and KLK10), which are proteases considered as important prognostic factors of various cancers [168]. Its inhibitory potency has also been examined towards cockroach extract (CRE) trypsin-like protease (TLP) activity [255]. It turned out that PE-BBI is a potent inhibitor of TLP, but, interestingly, it does not inhibit the activity of the host airway TLP (assay on human bronchial epithelial cell line 16HBE). Notably, it also ameliorates cockroach-induced cell damage (16HBE cell line). Inhibition of cockroach TLP might have therapeutic potential in the treatment of cockroach allergy. PE-BBI inhibits CRE and, in effect, protects airway epithelial cultures from cell damage and barrier disruption caused by CRE. In contrast, such a promising result has not been observed for pLR-HL, which inhibits CRE with similar potency but, even despite sharing identical disulfide bridge loop and similar inhibitory potency towards trypsin, inhibits also host TLP.

## 6. Conclusions

BBIs’ intrinsically high inhibitory activity combined with extreme thermal, proteolytic, and pH stability build the fundaments of their potential for diverse applications. Even though the classical soybean BBI does not meet high initial expectations to become an effective, natural anticancer agent, it is shown that it might be considered as a complement for other molecules endowed with more evident anti-cancer properties, such as *α*-tocopheryl succinate or bioactive peptide lunasin.

Noteworthy, BBIs’ biomedical application in the treatment of various diseases related to dysregulated proteolytic activity, not only cancers but also metabolic and inflammatory disorders, is still under examination.

Moreover, various BBIs may be utilized as efficient tools for learning the exact role of proteolytic enzymes involved in diseases’ progress and development. They are also attractive starting structures for designing novel, potent, synthetic inhibitors and other compounds, displaying a combination of various capabilities. The later merit has been shown for SFTI-1, in which simultaneous rational modifications of both loops have resulted in novel bifunctional bioactive peptides. In the case of some BBIs and BBLTIs, this unique combination of strong inhibitory activity towards proteolytic enzymes with bactericidal potency and low toxicity may result in novel antimicrobial agents. In the light of growing antibiotic resistance and the high propensity of known antimicrobial peptides to hydrolytic breakdown, such compounds seem to be of particular interest.

Despite the physiological role of BBIs in plants and animals is still vague, it is their multifaceted biological activity that draws a lot of researchers’ attention.

## Figures and Tables

**Table 1:** The most representative plant BBIs, their origin, inhibitory activities, and PDB codes (if available) of their crystal and/or solution structures.

Source and Name	Inhibitory Activity Expressed as *K*_i_ (nM) (If not Stated Otherwise) (Enzyme); PDB Code
*Glycine max* (Soybean) Bowman-Birk inhibitor (various isoinhibitors) **BBI**	ranged from 3.2 to 29.8 (trypsin) [65,66]; 3.3 (chymotrypsin) [66]; IC_50_ 20 μM (proteasome ChT-L) [67]. Crystal structure with trypsin 1K9B [68]; ternary complex with trypsin 1D6R [62]; crystal structure with chymotrypsin 5J4Q; solution structure [69].
*Vigna unguiculata* (Black-eyed pea) trypsin and chymotrypsin inhibitor **BTCI**	20 (trypsin) and 0.42 (trypsin, using surface plasmon resonance); 120 (chymotrypsin) and 0.41 (chymotrypsin, using surface plasmon resonance) [63]; 100 (proteasome T-L); 700 (proteasome ChT-L); 1400 (proteasome C-L) [60]. Crystal structure 2R33 [53], structure with trypsin 2G81 [63], structure with trypsin and chymotrypsin 3RU4 [70]
*Geoffroea decorticans* trypsin inhibitor **GdTI**	2.1 (trypsin) [71]; 0.18 μM (IC_50_, α-glucosidase) [71]
*Apios americana* trypsin inhibitor **AATI**	3 (trypsin); 1000 (chymotrypsin) [72]
Inhibitor from *Lupinus albus* (White lupin)	4.2 (trypsin) [73]
*Luetzelburgia Auriculata* ((Allemao) Ducke) Bowman-Birk inhibitor **LzaBBI**	0.86 (trypsin); 1.2 (chymotrypsin) [74]
Inhibitors from *Cajanus cajan* (Red gram)	292 (trypsin); 2265 (chymotrypsin) [75] 272 (trypsin); 3725 (chymotrypsin) [76]
*Dolichus biflorus* Bowman-Birk inhibitor	40 (trypsin); 480 (chymotrypsin) [77]
*Vigna mungo* (Black gram) protease inhibitor **BgPI**	309.8 (trypsin); 10,770 (chymotrypsin) [78]
Twelve *Lathyrus sativus* Bowman-Birk isoinhibitors **Ls_BBI**	ranged from 6.9 to 30.8 (trypsin); ranged from 11.7 to 26.0 (chymotrypsin); Ls_BBI3c 54.6 (elastase) [79]
*Phaseolus acutifolius* (Tepary bean) protease inhibitor **TBPI**	280 (trypsin); 68 (chymotrypsin) [80]
*Clitoria fairchildiana* (Sombreiro) protease inhibitor **CFPI**	0.33 (trypsin); 0.15 (chymotrypsin) [81]
*Dioclea glabra* trypsin inhibitor **DgTI**	0.5 (trypsin) [82]
*Vicia faba* (Faba bean) trypsin inhibitor **VFTI-G1**	20.4 (trypsin) [83]
*Pisum sativum* (Winter peas) trypsin isoinhibitors **PsTI**	ranged from 1.2 to 0.84 (trypsin); ranged from 21 to 15 (chymotrypsin) [84]; crystal structure 1PBI [59]
*Rhynchosia sublobata* Bowman-Birk inhibitors **RsBBI**	128.5 (trypsin); 807.8 (chymotrypsin) [55]
*Lens culinaris* (Lentil) trypsin inhibitor **LCTI**	0.54 (trypsin); 7.25 (chymotrypsin). Solution structure 2AIH [85]
*Cratylia mollis* (Camaratu bean) trypsin inhibitor **CmTI_2_**	1.4 (trypsin) [86]
*Medicago scutellata* (Snail medic) trypsin inhibitor **MsTI**	1.8 (trypsin) [87]. Crystal structure with trypsin 2ILN [88].
*Torresea cearensis* trypsin inhibitor **TcTI**	1 (trypsin); 36 (plasmin); 50 (chymotrypsin); 1450 factor XIIa [89]

ChT-L—chymotrypsin-like activity; C-L—caspase-like activity; T-L—trypsin-like activity.

**Table 2:** SFTI-1 and its synthetic analogs, their inhibitory activities, and PDB codes (if available) of their crystal and/or solution structures. Modified positions are marked in bold. The cyclization is indicated by (&), according to the recommendation of Spengler et al. [90].

SFTI-1 from *Helianthus annuus* and Its Synthetic Analogs	
Name (If Given) and Sequence	*K*_i_ (nM) (Enzyme) (Structure in PDB, If Reported)
**Bicyclic(native) SFTI-1** &^1^GRC(&^2^)TKSIPPIC(&^2^)FPD&^1^	0.1 (trypsin) [49] (crystal structure 1SFI [49], 1JBL solution structure [91]); 102 (matriptase) (crystal structure with matriptase catalytic domain 3P8F [92]); 218 (matriptase-2) [93]; 0.15 (cathepsin G); 105,000 (elastase); 7400 (chymotrypsin); 136,000 (thrombin) [49]; 143 (KLK5); 25.1 (KLK14) [94]; 4960 (mesotrypsin) [95]
**Monocyclic SFTI-1** GRC(&)TKSIPPIC(&)FPD	0.02698 (trypsin) [96] (solution structure 1JBN [91]);703 (matriptase) [97]; 61 (matriptase); 1365 (matriptase-2) [93]; 26,980 (20S proteasome, ChT-L); 29,090 (20S proteasome, C-L) [98]
GRC(&)TKSI**A**PIC(&)FPD	27 (matriptase); 0.035 (trypsin) [99]
GRC(&)TKSIP**A**IC(&)FPD	370 (matriptase); 0.0017 (trypsin) [99]
GRC(&)TKSIPPIC(&)F**A**D	240 (matriptase); 0.0037 (trypsin) [99]
GRC(&)TKSIPPIC(&)FP**A**	1500 (matriptase); 0.01 (trypsin) [99]
&^1^GRC(&^2^)T**R**SIPPIC(&^2^)FPD&^1^	19 (matriptase-2); 269 (matriptase); 13.4 (trypsin) [93]
GRC(&)T**R**SIPPIC(&)FPD	91 (matriptase); 115 (matriptase-2); 15.2 (trypsin) [93]
GRC(&)T**F**SIPPIC(&)FPD	0.5 (chymotrypsin) [100]
GRC(&)T**X**SIPPIC(&)FPD **X** = 4-fluoro-l-phenylalanine	0.03 (chymotrypsin) [101] (*K*_i_ calculated as a reciprocal value of originally published *K*_a_ 3.0 × 10^10^ M^−1^)
GRC(&)T**V**SIPPIC(&)FPD	71 (neutrophil elastase) [102]
GRC(&)TKSIPP**R**C(&)FPD	6.4 (matriptase); 0.0038 (trypsin) [99]
GRC(&)TKSIPP**K**C(&)FPD	40 (matriptase); 0.0057 (trypsin) [99]
G**K**C(&)TKSIPPIC(&)FPD	1200 (matriptase); 0.002 (trypsin) [99]
GRC(&)T**R**SIP**X**IC(&)FPD **X** = Abu (aminobutyric acid)	0.5 (trypsin) [100]
&^1^G**r**C(&^2^)T**R**SIPPIC(&^2^)FPD&^1^ **r** = d-Arg	280 (matriptase-2); 63,360 (matriptase) [93]
G**r**C(&)T**R**SIPPIC(&)FPD **r** = d-Arg	433 (matriptase-2); 76,310 (matriptase) [93]
&^1^**K**RC(&^2^)T**R**SIPPIC(&^2^)FPD&^1^	127 (matriptase-2); 532 (matriptase) [103]
&^1^GRC(&^2^)TKSIPP**R**C(&^2^)**H**PD&^1^	3.6 (matriptase) [97]
**SDMI-1** GRC(&)TKSIPP**R**C(&)**H**PD	11.2 (matriptase) [97]
**SDMI-3** KRC(&)TKSIPPRC(&)HPD	2.1 (matriptase) [97]
&^1^**K**RC(&^2^)TKSIPP**R**C(&^2^)**H**PD&^1^	4.1 (matriptase) [97]
**K**(&^1^)RC(&^2^)TKSIPP**R**C(&^2^)**H**PD&^1^	4.1 (matriptase) [97]
**K**(&^1^)RC(&^2^)TKSIPP**R**C(&^2^)**H**P&^1^	7.2 (matriptase) [97]
**K**(&^1^)RC(&^2^)TKSIPP**R**C(&^2^)**H**&^1^	2.6 (matriptase) [97]
&^1^GRC(&^2^)T**R**SIPP**R**C(&^2^)**H**PD&^1^	15 (matriptase-2); 4.9 (matriptase) [103]
GRC(&)T**R**SIPP**R**C(&)**H**PD	127 (matriptase-2); 532 (matriptase) [103]
&^1^**K**RC(&^2^)T**R**SIPP**R**C(&^2^)**H**PD&^1^	102 (matriptase-2); 8.3 (matriptase); 9 (trypsin) [103]
**K**(&^1^)RC(&^2^)T**R**SIPP**R**C(&^2^)**H**PD&^1^	257 (matriptase-2); 2.6 (matriptase); 5.1 (trypsin) [103]
**K**RC(&)T**R**SIPP**R**C(&)**H**PD	318 (matriptase-2); 4.3 (matriptase); 5.3 (trypsin) [103]
&^1^GRC(&^2^)T**R**SIPP**H**C(&^2^)**W**PD&^1^	51 (KLK5) [104]
&^1^GRC(&^2^)T**R**S**Y**PPIC(&^2^)FPD&^1^	214 (thrombin) [105]
G**V**C(&)T**L**SIPPIC(&)FPD	300 (pancreatic elastase) [106]
&^1^GRC(&^2^)**Y**KS**K**PPIC(&^2^)FPD&^1^	0.05 (plasmin, crystal structure 6D3X); 160 (trypsin); 29,000 (cathepsin G) [107]
&^1^GRC(&^2^)**QX**S**E**PP**E**C(&^2^)FPD&^1^ **X**= 4-chloro-l-phenylalanine	1.8 (chymase); 330 (chymotrypsin); 150 (cathepsin G) [108]
&^1^G**T**C(&^2^) **X_1_** **X_2_**S**D**PPIC(&^2^)FP**N**&^1^ **X_1_** = norleucine; **X_2_** = 4-guanidine-l-phenylalanine	1.6 (cathepsin G) [109]
GRC(&)T**X**SIPPIC(&)FPD **X** = 4-guanidine-l-phenylalanine	5.55 (chymotrypsin) [101] (*K*_i_ calculated as a reciprocal value of originally published *K*_a_ 1.8 10^8^ M^−1^)
**K**RC(&)**K**KSIPP**R**C(&)**H**PD	3.8 (furin) [110]
**K**RC(&)**K**KSIPP**R**C(&)F-NH_2_	0.49 (furin) [110]
&^1^G**F**C(&^2^)**QR**SIPPIC(&^2^)FPD&^1^	3.59 (KLK4 [111], crystal structure 4K1E [112])
&^1^G**F**C(&^2^)**QR**SIPPIC(&^2^)FP**N**&^1^	0.04 (KLK4 [90], crystal structure 4KEL [113])
&^1^G**Y**C(&^2^)**NR**S**Y**PP**E**C(&^2^)FP**N**&^1^	0.34 (KLK5); 18 (KLK14) [114]
&^1^G**F**C(&^2^)**HR**S**Y**PP**E**C(&^2^)**W**P**N**&^1^	2.4 (KLK5, solution structure 6NOX) [114] 150 (KLK14) [114]
&^1^G**K**C(&^2^)**LF**S**N**PPIC(&^2^)FP**N**&^1^	0.14 (KLK7); 170 (chymotrypsin) [115]
&^1^G**W**C(&^2^)**IR**S**K**PPIC(&^2^)**N**P**N**&^1^	7.0 (KLK14); 19.9 (KLK4); 3200 (trypsin) [105]
**SFMI-1** G**I**C(&)**SR**S**L**PPIC(&)**I**PD	65 (MASP-1); 1030 (MASP-2); 260 (trypsin) [116]
**SFMI-2** G**Y**C(&)**SR**S**Y**PP**V**C(&)**I**PD	180 (MASP-2); 1000 (trypsin) [116]
GRC(&)T**R**S**X**PPIC(&)FPD **X** = 4,4′-biphenyl-l-alanine	28 (mesotrypsin) [117]
&^1^G**X_1_**C(&^2^)**YX_2_**S**Y**PPIC(&^2^)**N**P**N**&^1^ **X_1_** = 4,4′-biphenyl-l-alanine; **X_2_** = norvaline	6.1 (proteinase 3); 16 (neutrophil elastase) [118]
&^1^G**T**C(&^2^)**YX**S**Y**PPIC(&^2^)**N**P**N**&^1^**X = Abu**	7.0 (proteinase 3); 3.2 (neutrophil elastase) [118]
GRC(&)T**R**S**KK**PIC(&)FPD	310 (20S proteasome, ChT-L); 20,140 (20S proteasome, T-L); 680 (20S proteasome, C-L) [96]
&^1^G**T**C(&^2^)T**R**SIPPIC(&^2^)**N**P**N**&^1^	0.71 (trypsin [94], crystal structure 6BVH [119])
**RX**C(&)T**R**S**KK**PIC(&)FPD **X** = *N*-arginine (peptoid monomer)	80 (20S proteasome, ChT-L); 14,000 (20S proteasome, T-L); 140 (20S proteasome, C-L) [98]
**O2Oc**-GRC(&)T**R**S**KK**PIC(&)FPD	310 (20S proteasome, ChT-L); 1150 (20S proteasome, T-L); 350 (20S proteasome, C-L) [98]
RGC(&^1^)TRSKKPIC(&^2^)GPGGGC(&^2^)TR--SKKPIC(&^1^)FPD	30 (20S proteasome, ChT-L); 20 (20S proteasome, C-L) [98]

SFTI-1—Sunflower trypsin inhibitor 1; SDMI—SFTI-1 derived matriptase inhibitor -1; SFMI- sunflower mannose-binding lectin-associated serine protease inhibitor; ChT-L—chymotrypsin-like activity; C-L—caspase-like activity; T-L—trypsin-like activity; KLK—kallikrein; MASP-1—mannan-binding lectin serine protease 1; MASP-2—mannan-binding lectin serine protease 2; Abu-aminobutyric acid; O2Oc—8-amino-3,6-dioxaoctanoyl group.

**Table 3:** Anti-cancer properties of BBIs and BBLTIs.

Inhibitor Source	Type of Cancer	Cell Line and/or Animal Model/Reference	The Observed Effect
**Plant-Derived BBIs**			
*Glycine max* (Soybean) Bowman-Birk inhibitor **BBI** (or BBIC)	colorectal	HT29 cell line [66]	Inhibited proliferation at concentrations ranged from 31 μM to 125 μM, cell cycle arrest in the G0–G1 phase.
		DMH-induced colon cancer in rat [138]	Suppressive effect on colon carcinogenesis. Diet supplemented with 0.1% and 0.5% of inhibitor.
		dextran sulfate sodium-induced ulcerative colitis in mice [139]	Reduction of inflammation, lower mortality rate, delayed onset of mortality. Diet supplemented with 0.5% of inhibitor.
		DMH-induced colorectal neoplasia in *Swiss* mice [140]	Protection from inflammatory processes and from the appearance of pre-malignant lesions. Diet supplemented with 0.1% of inhibitor.
		DMH-induced colon carcinogenesis in mice [141]	Reduction of the incidence of adenocarcinomas of the colon by ∼50%. Diet supplemented with 0.1% of inhibitor.
	prostate	LNCaP prostate cancer xenograft mouse model [142]	Suppressive effect on the tumor growth in nude mice and an increase of the serum PSA concentration. Diet supplemented with 0.1% of inhibitor.
		various normal and cancer cell lines, including LNCaP and PC-3 [143]	Cell growth inhibition at the concentration of 100 μg/mL;BBIC inhibited clonogenic survival.
		patients with benign prostatic hyperplasia and lower urinary tract symptoms [144]	Phase I clinical trial.
		*N*-methyl-*N*-nitrosourea + testosterone-induced prostate carcinogenesis in rats [145]	Inhibition of induced prostate carcinogenesis in the Wistar-Unilever rats. BBIC administered at 200 or 2000 mg/kg diet dose.
		LNCaP cell line and LNCaP stem-like cells [146]	Combination of BBI and *α*-tocopheryl succinate results in cell growth inhibition and induction of apoptosis. BBI at the concentration of 200 μg/mL.
		LNCaP cell line and the transgenic rats developing adenocarcinoma of the prostate [147]	Induction of Cx43 expression and apoptosis at the concentration of 500 μg/mL. Reduced progression of adenocarcinomas in the lateral prostate lobes in rats.
	ovarian	A2780 cell line and its cisplatin-resistant sublines C30, C200 [148]	Suppression of the clonogenic cells survival and a boost of cisplatin-induced growth inhibition and/or cytotoxicity at the concentrations of 50 and 100 μg/mL.
		M5076 sarcoma xenograft mouse model [149,150]	Reduction of relative tumor weight associated with induced expression of Cx43. Diet supplemented with 0.5% BBI.
	breast	MCF7 cell line [67,151,152]	Decreased clonogenic survival of cells at the concentration 100 μg/ml [151], with IC_50_ of about 35 μM [152]; downregulation of cyclin D1 and E1, upregulation of mitogen-activated protein kinase phosphatase 1 (MKP-1), and suppression of phosphorylated extracellular signal-related kinases (ERK1/2) activity upon treatment with 20 μM [67].
		xenograft model of nude mice transplanted with MDA-MB-231 cells [153]	BBI injection at 20 mg/kg body weight shows no effect on tumor incidence. BBI protects lunasin, an actual bioactive agent, from digestion.
	oral leukoplakia	patients with oral leukoplakia [154,155]	Phase IIa clinical trial.
	oral cavity	DMBA-induced oral carcinogenesis in hamster [156]	Suppression of the carcinogenesis at the concentrations ranging from 1% to 0.01%.
	head and neck carcinoma	SCC61 cell line [151]	Suppression of the clonogenic survival of cell line and enhancement of radiation-induced cell killing at the concentration of 100 μg/mL.
	hepatic	HepG2 cell line [152]	Inhibited proliferation with IC_50_ of about 140 μM.
	liver	DMH-induced liver carcinogenesis in mice [157]	Suppression of the DMH-induced carcinogenesis in the mouse liver and gastrointestinal tract. Diet supplemented with 0.5% and 0.1% inhibitor.
	osteosarcoma	U2OS cell line [158]	Cell growth inhibition, induction of Cx43, induction of apoptosis at the concentration 200 μg/ml; BBI-dependent negative growth control was based on cytostatic and cytotoxic effects.
	leukemia	L1210 cell line [159]	Cell growth inhibition with IC_50_ of about 22.5 μM.
*Vigna unguiculata* (Black-eyed pea) trypsin and chymotrypsin inhibitor **BTCI**	breast	MCF-7 and/or MDA-MB-231 cell lines [160,161]	Cell growth inhibition, cytostatic effect at the G2/M phase, induction of apoptosis at the concentrations 100 μM (MDA-MB-231).
*Phaseolus vulgaris* (Kidney bean) Bowman-Birk inhibitor	breast	MCF7 cell line [162]	Inhibited proliferation with IC_50_ of about 71.5 μM.
	prostate	LNCaP cell line [163]	Inhibited proliferation at the concentrations 200, 400 μg/mL.
*Vigna radiata* (Mungbean) Bowman-Birk inhibitor	prostate	LNCaP cell line [163]	Inhibited proliferation at the concentrations 100, 200 μg/mL.
*Cicer arietinum* (Chickpea) Bowman-Birk inhibitor	prostate	PC-3 and LNCaP cell lines [163]	Inhibited proliferation at the concentrations 25–400 μg/mL.
	breast	MDA-MB-231 cell line [163]	Inhibited proliferation at the concentrations 25–400 μg/mL.
*Pisum sativum* (Pea) trypsin inhibitor **TI1B**	colorectal	HT29 cell line [164,165]	Inhibited proliferation with IC_50_ ranged from 32 μM (rTI1B) to 73 μM (rTI2B).
*Vicia faba* (Faba bean) trypsin inhibitor **VFTI-G1**	hepatoma	HepG2 cell line [83]	Inhibited proliferation with IC_50_ of about 30 μM; induced chromatin condensation and cell apoptosis.
*Lens culinaris* (Lentil) Bowman-Birk inhibitor	colorectal	HT29 cell line [166]	Inhibited proliferation with IC_50_ of about 32 μM.
*Macrotyloma axillare* (Horsegram) Bowman-Birk inhibitor	colorectal	DMH-induced colorectal neoplasia in *Swiss* mice [140]	Protection from inflammatory processes and the appearance of pre-malignant lesions. Diet supplemented with 0.1% of inhibitor.
**Animal-Derived BBLTIs**			
The skin secretion of Asian green frog, *Hylarana erythraea*	prostate	PC-3 cell line [167]	Inhibited proliferation at the concentration 1 mM.
	lung	H157 cell line [167]	Inhibited proliferation at the concentration 1 mM.
	breast	MCF-7 cell line [167]	Inhibited proliferation at the concentration 1 mM.
The skin secretion of frog *Pelophylax esculentus*	colorectal	DLD-1, DKS8, HCT116, and HKE3 cell lines [168]	Inhibited proliferation with IC_50_ 50.1 µM, 9.8 µM, 35.4 µM, 50.2 µM, respectively.
The skin secretions of *Pelophlax plancyi fukienesis* (chimeric peptide called Tat-loop)	lung cancer	H460, H157 [32]	Inhibited proliferation at the concentration 100 µM.
The skin secretions of *Sylvirana latouchii* (F-SL analog)	human non-small cell lung cancer (NSCLC)	H157, H460, H838, and H23 [169]	Induced caspase 3/7 activation, which confirms induced apoptosis in H157 (IC_50_ of about 101.4 µM) and H838 (IC_50_ of about 59.74 µM).
	breast	MCF-7 [169]	Inhibited proliferation with IC_50_ of about 201.7 µM.
	prostate	PC-3 [169]	Inhibited proliferation with IC_50_ of about 158.6 µM.

PSA—prostate-specific antigen; DMH—1,2-dimethylhydrazine; DMBA—7,12-dimethyl-benz[a]anthracene.

**Table 4:** Inhibitory activities expressed as K_i_ values and antimicrobial properties expressed as MIC or lethal concentration (LC) values of amphibian-derived BBLTIs and their most important analogs. Modified positions are marked in bold.

Origin	Name (If Given) and Sequence	*K*_i_ (nM) (Enzyme)	Antimicrobial Potency (If Determined)
*Lithobates pipiens*, (formerly *Rana pipiens*) (Northern Leopard frog) [208]	**pLR** LVRGC(&)WTKSYPPKPC(&)FVR	110 (trypsin) [208]	*E. coli*, *Y. pseudotuberculosis*, *Ps. syringae* pv *tabaci* (LC >100 μM); *B. magaterium* (LC 20 μM); *S. lentus* (LC 50 μM); *M.luteus* (LC 10 μM); *C. albicans* (LC >100 μM); *C. tropicalis* (LC 11 μM); *C. guiller-mondii* (LC 6.6 μM); *P. nicotianae spores* (LC 75 μM) [244]
	&^1^LVRGC(&^2^)WTKSYPPKPC(&^2^)FVR&^1^	70 (trypsin) [208]	Not studied
	LVRGC(&)WTKSYPPKPC(&)	322 (trypsin) [208]	Not studied
	C(&)WTKSYPPKPC(&)	335 (trypsin) [208]	Not studied
*Lithobates capito* (formerly *Rana sevosa*) (Dusky Gopher frog) [209]	**pYR** YLKGC(&)WTKSYPPKPC(&)FSR		Not studied
*Rana esculenta* (Common Water Frog) [244]	**Ranacyclin E** SAPRGC(&)WTKSYPPKPC(&)K-NH_2_	129 (trypsin) [208]	*E. coli* (LC >100 μM); *Y. pseudotuberculosis* YP III (LC 9 μM); *Ps. syringae* pv *tabaci* (LC 80 μM); *B. magaterium* (LC 3 μM); *S. lentus* (LC 7 μM); *M.luteus* (LC 5 μM); *C. albicans* (LC >100 μM); *C. tropicalis* (LC 7.4 μM); *C. guiller-mondii* (LC 3.4 μM); *P. nicotianae spores* (LC 32 μM) [244]
*Rana temporaria*(European common frog) [244]	**Ranacyclin T** GALRGC(&)WTKSYPPKPC(&)K-NH_2_	116 (trypsin) [208]	*E. coli* D21 (LC 30 μM); *Y. pseudotuberculosis* YP III (LC 5 μM); *Ps. syringae* pv *tabaci* (LC 16 μM); *B. magaterium* (LC 3 μM); *S. lentus* (LC 10 μM); *M.luteus* (LC 7 μM); *C. albicans* (LC 22 μM); *C. tropicalis* (LC 14 μM); *C. guiller-mondii* (LC 1.0 μM); *P. nicotianae spores* (LC 16 μM) [244]
*Odorrana graham* [31] (Garaham’s frog)	**ORB** AALKGC(&)WTKSIPPKPC(&)FGKR	3.06 × 10^5^ (trypsin) [31]	*E. coli* (MIC 3.20 µg/ml); *S. aureus* (MIC 5.83 µg/ml); *B. subtilis* (MIC 1.85 µg/ml); *C. albicans* (MIC 2.40 µg/ml) [31]
	**ORB2** LKGC(&)WTKSIPPKPC(&)FG	685 (trypsin) [31]	No antimicrobial activity (*E. coli,* *S. aureus, B. subtilis, C. albicans)* [31]
	**ORB1** LKGC(&)WTKSIPPKPC(&)F	4 × 10^6^ (trypsin) [31]	*E. coli* (MIC 2.34 µg/ml); *S. aureus* (MIC 1.76 µg/ml); *B. subtilis* (MIC 2.34 µg/ml); *C. albicans* (MIC 4.69 µg/ml) [31] No antimicrobial activity at concentration range up to 250 mg/ml (*S. aureus, S. epidermidis, B. subtilis, B. cereus, E. coli, P. aeruginosa*) [248]
	**ORB-CF** C(&)WTKSIPPKPC(&)F	2.2 × 10^6^ (trypsin) [31]	*E. coli* (MIC 8.90 µg/ml); *S. aureus* (MIC 5.96 µg/ml); *B. subtilis* (MIC 10.50 µg/ml); *C. albicans* (MIC 19.4 µg/ml) [31]
	**ORB-C** C(&)WTKSIPPKPC(&)	710 (trypsin) [31]	No antimicrobial activity (*E. coli,* *S. aureus, B. subtilis, C. albicans)* [31]
	**ORB2-K** LKGC(&)WTKSIPPKPC(&)FGK	3 (trypsin) [250]; 886 (trypsin) [31]	No antimicrobial activity (*E. coli,* *S. aureus, B. subtilis, C. albicans)* [31]
*Huia versabilis* (Bamboo Leaf Odorous Frog) [249]	**HV BBI** SVIGC(&)WTKSIPPRPC(&)FVK-NH_2_	18.8 (trypsin) [249]; 120 (trypsin) [248]; 155 (matriptase-1) [248]; 82 (plasmin) [248]	*S. aureus* (MIC 60 µg/ml at Davis Minimal Broth, no antimicrobial activity at Mueller–Hinton Broth) [251]
	**[Arg^8^]HV-BBI** SVIGC(&)WT**R**SIPPRPC(&)FVK-NH_2_	54.2 (trypsin) [249]	Not studied
	**[Phe^8^]HV-BBI** SVIGC(&)WT**F**SIPPRPC(&)FVK-NH_2_	389 (chymotrypsin) [249]	No antimicrobial activity (*E. coli,* *S. aureus*) [251]
	**HV BBI (4-16)** GC(&)WTKSIPPRPC(&)F-NH_2_	8 (matriptase-1) [248]; 151 (trypsin) [248]	Not studied
	**HV-BBI (3-18)** IGC(&)WTKSIPPRPC(&)FVK-NH_2_	4 (trypsin) [251]	*E. coli* (MIC 160 µg/ml); *S. aureus* (MIC 80 µg/ml) (at Davis Minimal Broth, no antimicrobial activity at Mueller–Hinton Broth) [251]
*Odorrana hejiangensis* [253] (Hejiang Odorous Frog)	**HJTI** GAPKGC(&)WTKSYPPQPC(&)S	388 (trypsin) [253]	No antimicrobial activity (at concentrations up to and including 180 M; *E. coli, S. aureus,* and *C. albicans*) [253]
	**[Lys^14,17^]HJTI** GAPKGC(&)WTKSYPP**K**PC(&)**K**	217 (trypsin) [253]	*E. coli* (MIC 160 μM)
*Hylarana latouchii*(Broadfolded Frog) [252]	**pLR-HL** LIGGC(&)WTKSIPPKPC(&)LV	143 (trypsin) [252]	No antimicrobial activity (*E. coli, S. aureus* and *C. albicans*) [252]
	LIGGC(&)WTFSIPPKPC(&)LV	2141 (chymotrypsin) [252]	Not studied
*Odorrana schmackeri* (Piebald Odorous Frog) [254]	**OSTI** AALKGC(&)WTKSIPPKPC(&)F-NH_2_	0.3 (trypsin) [254]; 2500 (tryptase) [254]	Not studied
	**[Phe^9^]OSTI** AALKGC(&)WT**F**SIPPKPC(&)F	1000 (chymotrypsin) [254]	Not studied
*Hylarana erythraea* (Asian Green Frog) [167]	**HECI** TVLRGC(&)WTFSFPPKPC(&)I-NH_2_	3920 (chymotrypsin) [167]; 8550 (proteasome 20S C-L) [167]	Not studied
*Pelophylax esculentus* [168] (Green Frog)	**PE-BBI** GALKGC(&)WTKSIPPKPC(&)K-NH_2_	310 (trypsin) [168]	No antimicrobial activity [168]
*Pelophlax plancyi* *Fukienesis* [32] (Fukien Gold-Striped Pond Frog)	**PPF-BBI** ALRGC(&)WTKSIPPKPC(&)P-NH_2_	170 (trypsin) [32]; 30,730 (tryptase) [32]	*E. coli* (MIC 128 μM); *S. aureus* (MIC 128 μM); MRSA (MIC 512 μM); *P.aureginosa* (MIC > 512 μM); *C. albicans* (MIC > 128 μM) [32]
	**[Phe^8^]PPF-BBI** ALRGC(&)WT**F**SIPPKPC(&)P-NH_2_	850 (chymotrypsin) [32]	*E. coli* (MIC > 512 μM); *S. aureus* (MIC > 512 μM); MRSA (MIC > 512 μM); *P.aureginosa* (MIC > 512 μM); *C. albicans* (MIC > 512 μM) [32]
	**[Pro^16^]PPF-BBI** ALRGC(&)WTKSIPPKPC(&)**K**-NH_2_	112 (trypsin) [32]	*E. coli* (MIC 128 μM); *S. aureus* (MIC 64 μM); MRSA (MIC > 512 μM); *P.aureginosa* (MIC 512 μM); *C. albicans* (MIC 128 μM) [32]
	**Tat-loop** **RKKRRQRRR**C(&)WTKSIPPKPC(&)	607 (trypsin) [32]	*E. coli* (MIC 128 µM); *S. aureus* (MIC 128 µM); MRSA (MIC 256 µM); *P.aureginosa* (MIC 256 µM); *C. albicans* (MIC 4 μM) [32]
*Pelophylax nigromaculatus* (East AsianFrog) [30]	**Ranacyclin NF (RNF)** GAPRGC(&)WTKSYPPQPC(&)F-NH_2_	447 (trypsin) [30]; 6774 (tryptase) [30]	*S. aureus* (MIC 512 µM) [30]
	**RNF1** GAPRGC(&)WTKSYPPQPC(&)F	1300 (trypsin) [30]; 9059 (tryptase) [30]	No antimicrobial activity [30]
	**RNF3L** GALRGC(&)WTKSYPPQPC(&)F-NH_2_	201 (trypsin) [30]; 12,500 (tryptase) [30];	No antimicrobial activity [30]
